# From Regenerative Mechanisms to Clinical Practice: Current Status, Controversies, and Future Perspectives of Platelet-Rich Plasma in Urology and Sexual Medicine

**DOI:** 10.3390/jcm15082949

**Published:** 2026-04-13

**Authors:** Rui Qu, Jiaqi Gu, Yi Luo, Luo Yang, Yi Dai

**Affiliations:** 1 Department of Urology and Pelvic Surgery and Andrology, West China School of Public Health and West China Fourth Hospital, Sichuan University, Chengdu 610041, China; 2 West China School of Public Health and West China Fourth Hospital, Sichuan University, Chengdu 610041, China

**Keywords:** platelet-rich plasma, erectile dysfunction, Peyronie’s disease, stress urinary incontinence, interstitial cystitis, bladder pain syndrome, urology, andrology, sexual medicine, regenerative therapy, reporting checklist

## Abstract

**Background/Objectives:** Platelet-rich plasma (PRP) is an autologous blood-derived biologic enriched in platelets and bioactive mediators. In urology and sexual medicine, PRP has been promoted for erectile dysfunction (ED) and a growing range of urogenital disorders on the premise that it may support angiogenesis, neuroregeneration, immune modulation, and tissue remodeling. However, clinical uptake has outpaced high-quality evidence, while heterogeneity in PRP preparation, characterization, and delivery limits interpretability and reproducibility. This structured narrative review aims to critically integrate mechanistic, preclinical, and clinical evidence regarding PRP use in ED, Peyronie’s disease (PD), stress urinary incontinence (SUI), interstitial cystitis/bladder pain syndrome (IC/BPS), and selected emerging indications. We further aim to identify sources of heterogeneity and propose an actionable minimum reporting framework (PRP-Uro Checklist) to guide future research. **Methods:** A structured search of PubMed/MEDLINE was conducted for studies published between 2021 and 2025. The relevant literature on PRP use in ED, PD, SUI, IC/BPS, and related indications was included for critical narrative synthesis. Emphasis was placed on PRP classification and preparation variables, outcome measure validity, and sources of heterogeneity across studies. **Results:** Mechanistic and preclinical evidence supports PRP’s potential to modulate nerve repair, angiogenesis, extracellular matrix remodeling, and immune polarization through a complex secretome of growth factors, cytokines, and extracellular vesicles (EVs). Clinical evidence suggests that intracavernosal PRP may improve erectile function in selected populations, but effect size, durability, and superiority over placebo remain uncertain due to small trials, substantial placebo effects, short follow-up, and incomplete biologic characterization. Evidence for PRP in PD, SUI, and IC/BPS remains preliminary and is derived largely from small cohorts, proof-of-concept studies, or uncontrolled designs, although early findings suggest potential symptom benefit and acceptable short-term tolerability. Across indications, inconsistent PRP reporting, particularly the absence of absolute platelet dose, leukocyte quantification, activation method, and standardized treatment protocols, represents a major barrier to reproducibility and evidence synthesis. **Conclusions:** PRP is biologically plausible and appears broadly safe, but its role in urology and sexual medicine remains investigational and is not yet supported by guideline-level evidence. To enhance reproducibility and interpretation, we propose a Minimum PRP Reporting Checklist for Urology and Sexual Medicine Trials (PRP-Uro Checklist). Future progress requires rigorous standardized reporting, indication-specific biologic characterization, rigorously designed sham-controlled trials, clinically meaningful endpoints, and longer-term follow-up.

## 1. Introduction

Erectile dysfunction (ED), Peyronie’s disease (PD), stress urinary incontinence (SUI), and interstitial cystitis/bladder pain syndrome (IC/BPS) impose substantial physical, psychological, and relational burdens. Although current therapies can be highly effective in selected patients, they often remain symptom-directed rather than restorative. For ED, phosphodiesterase type 5 inhibitors (PDE5is), intracavernosal vasoactive agents, vacuum erection devices, and penile prostheses can improve erectile performance mechanically or pharmacologically but do not directly reverse endothelial dysfunction, smooth muscle loss, fibrosis, or cavernous nerve injury, which underlie many cases, particularly after pelvic surgery or in cardiometabolic disease [1,2]. Similarly, PD has limited medical options with limited and variable effects on curvature; SUI is often managed with pelvic floor rehabilitation or surgery, but some patients seek less invasive alternatives; and IC/BPS remains heterogeneous, chronic, and often refractory to conventional stepwise therapies.

Against this clinical background, regenerative medicine approaches have gained increasing attention. PRP is an autologous blood-derived platelet concentrate intended to deliver a high local concentration of platelet-associated bioactive mediators. Platelets release growth factors and signaling molecules (e.g., platelet-derived growth factor (PDGF), vascular endothelial growth factor (VEGF), transforming growth factor-β (TGF-β), and insulin-like growth factor-1 (IGF-1)) that can coordinate angiogenesis, neurotrophic signaling, immune regulation, and extracellular matrix (ECM) remodeling [3]. In preclinical ED models, intracavernosal PRP has been associated with improved erectile parameters and attenuation of tissue injury in settings such as hyperlipidemia, diabetes, and cavernous nerve damage [4]. These data are mechanistically attractive; however, translation into clinical practice has been complicated by substantial variability in PRP formulations, preparation systems, activation methods, injection protocols, and outcome definitions. In addition to ED, exploratory applications have expanded toward PD, IC/BPS, recurrent urinary tract infections, and female pelvic floor disorders, but they are largely preliminary, relying on small cohorts or uncontrolled designs.

The central challenge is not only whether PRP “works”, but what PRP is in any given study. PRP products vary markedly by preparation system (single vs. double spin), platelet concentration, absolute platelet dose, leukocyte content, red blood cell contamination, activation strategy, fibrin architecture, and processing conditions. Such heterogeneity is widely recognized as a major barrier to interpreting PRP evidence across medical specialties [5,6]. A leukocyte-rich, activated product may behave differently from a leukocyte-poor, non-activated preparation, particularly when injected into highly specialized tissues such as the corpora cavernosa, tunica albuginea, bladder submucosa, or periurethral sphincteric complex. Yet many published studies report the injected volume without adequately characterizing the biologic product itself, thereby limiting reproducibility and meaningful comparison.

This problem is especially important in sexual medicine and functional urology, where placebo effects can be substantial, endpoints are often subjective, and commercial enthusiasm may outpace evidence. While previous systematic reviews have summarized the available literature, most have focused on individual indications, especially ED, and have not fully integrated mechanistic biology, product heterogeneity, and implementation-level reporting needs across the broader field.

This narrative review aims to (1) summarize the biological basis and proposed mechanisms of PRP relevant to urology and sexual medicine; (2) critically appraise preclinical and clinical evidence regarding PRP use in ED, PD, SUI, IC/BPS, and selected emerging indications; (3) identify the major sources of interpretive inconsistency, including product heterogeneity, delivery variability, endpoint selection, and patient phenotype; and (4) propose an actionable Minimum PRP Reporting Checklist for Urology/Sexual Medicine Trials (PRP-Uro Checklist). The goal is to provide not only a critical synthesis of current evidence, but also a practical framework to improve trial design, reporting quality, peer review, and future evidence synthesis in this rapidly expanding field. Unlike prior reviews that primarily summarize efficacy within a single indication, this article adopts a cross-indication translational perspective and treats PRP as an indication-specific biologic rather than a uniform intervention.

## 2. Methods

### 2.1. Review Design and Reporting Approach

This article was designed as a structured narrative review rather than a formal systematic review with exhaustive study inclusion or quantitative pooling. To strengthen rigor and transparency, this review was developed in accordance with the principles of the Scale for the Assessment of Narrative Review Articles (SANRA) and was informed by a structured PubMed/MEDLINE search strategy. The purpose was to provide a critical, cross-indication synthesis of the PRP literature in urology and sexual medicine, and address the broad conceptual issue of standardization in the field.

### 2.2. Search Strategy and Information Source

A structured search of PubMed/MEDLINE was performed for the literature published up to 31 October 2025. PubMed/MEDLINE was selected as the principal database because of its broad biomedical coverage and indexing of core journals in urology, andrology, sexual medicine, regenerative medicine, and translational science. The search strategy utilized combinations of keywords, including “platelet-rich plasma”, “PRP”, “erectile dysfunction”, “Peyronie’s disease”, “stress urinary incontinence”, “bladder pain syndrome”, “interstitial cystitis”, “urology”, “andrology”, and “sexual medicine”. Full search strings are provided in Appendix A.

### 2.3. Eligibility Criteria, Study Selection, and Prioritization

The eligible literature included (i) randomized controlled trials, comparative prospective studies, prospective single-arm clinical studies, and systematic reviews/meta-analyses involving PRP or closely related autologous platelet-derived biologics in urologic and sexual medicine indications; (ii) preclinical and translational studies directly relevant to nerve repair, angiogenesis, fibrosis/extracellular matrix remodeling, immunomodulation, or extracellular-vesicle-mediated mechanisms; and (iii) publication-date window (1 January 2021 to 31 October 2025).

Exclusion criteria were predefined as follows: non-English publications; duplicate datasets; non-autologous platelet products unless directly relevant to the conceptual discussion; reports without outcome data; and studies outside the review scope.

Because this review aimed at narrative synthesis rather than exhaustive inclusion of all clinically marginal or repetitive reports, studies selected for detailed discussion were further prioritized according to methodological informativeness, recency, relevance to current PRP controversies, adequacy of outcome reporting, and contribution to understanding product/protocol heterogeneity. Priority was given to sham- or placebo-controlled trials, prospective studies using validated outcomes, systematic reviews/meta-analyses, and mechanistic studies with direct translational relevance to current urologic practice: erectile dysfunction, Peyronie’s disease, stress urinary incontinence, and interstitial cystitis/bladder pain syndrome. Studies addressing adjacent genitourinary indications (e.g., genitourinary syndrome of menopause, lichen sclerosus, recurrent urinary tract infection, urethral reconstruction, infertility-related applications, and post-prostatectomy incontinence) were retained for contextual discussion but not included in the four-condition core evidence mapping.

**Figure 1:**
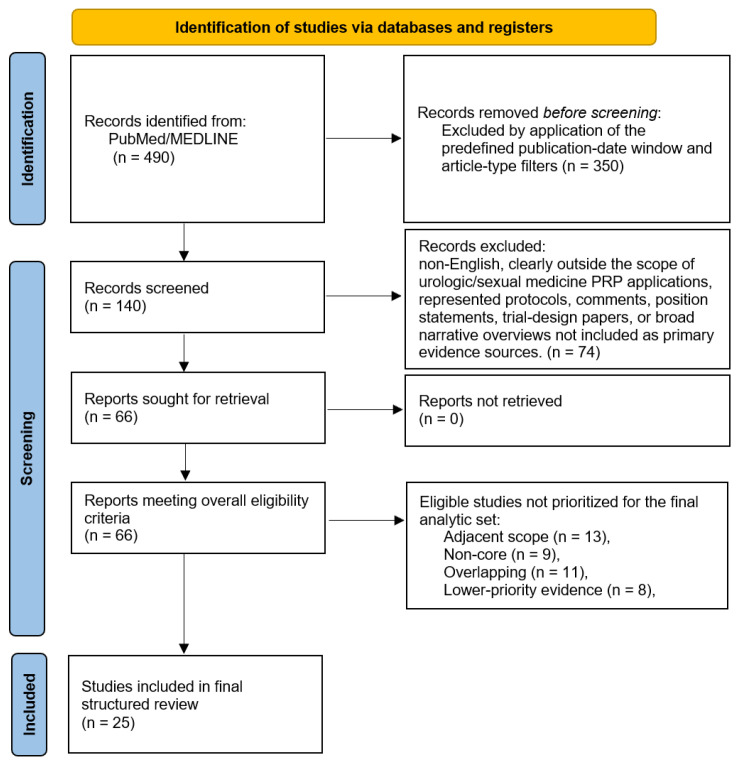
PRISMA flow diagram of the literature search and selection process. The diagram illustrates the number of records identified from PubMed/MEDLINE, screened, and finally included.

A total of 490 records were identified through database searching. After application of the predefined publication-date window (1 January 2021 to 31 October 2025) and article-type filters, 140 records remained (See Appendix B for filter details). These records were screened for inclusion in the primary evidence corpus. Seventy-four records were excluded because they were non-English, clearly outside the scope of urologic/sexual medicine PRP applications, or represented protocols, comments, position statements, trial design papers, or broad narrative overviews not retained as primary evidence sources. Sixty-six studies were retained in the overall eligible evidence corpus. Because the present review was intentionally structured around four predefined clinical domains, 41 eligible studies judged to be adjacent in scope, non-core to the disease-focused framework, overlapping, or lower-priority evidence were not retained in the final analytic set, and were used only for contextual background. The final structured narrative synthesis therefore included 25 studies addressing the four prespecified principal indications: erectile dysfunction, Peyronie’s disease, stress urinary incontinence, and interstitial cystitis/bladder pain syndrome. Given the narrative design, the PRISMA-style flow diagram (Figure 1) is presented as a transparent description of the screening process rather than as evidence of a fully systematic review.

### 2.4. Data Extraction and Synthesis Framework

For clinical studies, we extracted:

(1) PRP product variables: baseline whole-blood platelet count; final platelet concentration; absolute platelet dose delivered per session; leukocyte content; RBC contamination; activation method and timing; preparation protocol (single/double spin, RCF, time, device); handling/sterility (closed vs. open, time to injection).
(2) Delivery variables: route (intracavernosal, intraplaque, intravesical/submucosal, periurethral/urethral sphincter, intravaginal), injection mapping, imaging guidance, volume/session, number of sessions, interval, co-interventions (PDE5i, Li-ESWT).
(3) Outcomes: validated patient-reported outcomes (IIEF-EF/IIEF-5/EHS; PD curvature; SUI questionnaires/pad tests; IC symptom scores), objective endpoints when available (penile Doppler PSV/EDV with standardized stimulation), follow-up timepoints, and adverse event definitions.

Evidence was synthesized qualitatively, emphasizing:

(1) product/protocol heterogeneity;
(2) risk-of-bias vulnerabilities (blinding, allocation concealment, selective reporting);
(3) short vs. long-term outcomes;
(4) clinical meaningfulness.

### 2.5. Critical Appraisal and Risk-of-Bias Consideration

A formal RoB 2 or ROBINS-I assessment was not performed for all included studies because this manuscript was designed as a structured narrative review rather than a full systematic review. However, we conducted a structured critical appraisal of the major RCTs and prospective studies, focusing on domains most likely to influence interpretation in this field, including randomization, blinding integrity, comparator quality, co-intervention control, selective reporting, follow-up completeness, and adequacy of PRP characterization. Because internal validity is central to the interpretation of the ED literature, a pragmatic mini risk-of-bias appraisal is presented in the main text.

### 2.6. Artificial Intelligence Use Disclosure

Generative artificial intelligence tools (ChatGPT, version 5.2, OpenAI, San Francisco, CA, USA and DeepSeek, version V3.2, DeepSeek, Hangzhou, China) were used for limited language-related assistance during manuscript preparation, including grammar refinement, sentence-level clarity improvement, and formatting support. No artificial intelligence tool was used to generate scientific conclusions, determine study eligibility, perform data analysis, conduct critical appraisal, or develop the conceptual framework of the manuscript. All conceptualization, literature interpretation, critical synthesis, and checklist development were performed entirely by the human authors, who take full responsibility for the accuracy, originality, and integrity of the work.

## 3. PRP as a Biological Product: Definitions, Classifications, and Why Standardization Matters

### 3.1. What Constitutes PRP and Why the Label Alone Is Not Enough

PRP is often loosely defined as “plasma with a platelet concentration above baseline whole blood,” but this definition is insufficiently precise. For the purposes of this review, PRP refers to an autologous plasma-based platelet concentrate intended for liquid administration and characterized by platelet enrichment relative to whole blood, because its composition is variably influenced by platelet concentration, leukocyte content, red blood cell contamination, fibrin architecture, activation strategy, and processing protocol.

This definition should be distinguished from related but non-identical products such as platelet-rich fibrin (PRF), platelet-rich fibrin matrix (PRFM), and platelet lysates, which differ in fibrin architecture, handling characteristics, release kinetics, and potentially biological behavior.

This distinction matters because the literature in sexual medicine and regenerative urology sometimes groups these products together under the generic umbrella of “platelet-based therapies,” even though they may not be biologically interchangeable. If a study uses PRFM or a platelet lysate, its findings should not be assumed to represent injectable PRP unless the product characteristics and clinical context justify such interpretation.

Dohan Ehrenfest and colleagues proposed an influential classification distinguishing platelet concentrates according to leukocyte content and fibrin structure: pure PRP (P-PRP), leukocyte- and platelet-rich PRP (L-PRP), pure platelet-rich fibrin (P-PRF), and leukocyte- and platelet-rich fibrin (L-PRF) [7]. Subsequent systems (e.g., PAW-platelet, activation, white blood cell, PLRA-platelet, leucocyte, red blood cells, activation, DEPA-dose of platelet, efficiency, purity, activation, and MARSPILL method, activation, red blood cells, spin, platelets, image guidance, leukocytes, light activation) attempted to capture additional clinically relevant dimensions such as activation and dose [7]. The proliferation of classification schemes reflects a common recognition: “PRP” is not a single intervention, and the label itself is biologically under-informative unless key compositional features are specified.

From the perspective of urology and sexual medicine, this problem is not academic. ED, PD, IC/BPS, and pelvic floor disorders involve different tissue targets, cellular microenvironments, and dominant mechanisms of injury. A PRP product richer in leukocytes and inflammatory mediators may behave differently from a leukocyte-poor formulation, especially when injected into the corpora cavernosa or periurethral tissues. Yet many clinical trials incompletely report PRP composition, limiting the ability to compare results, reproduce protocols, or perform informative subgroup meta-analyses. Reviews across medical specialties consistently note inadequate reporting of PRP protocols and composition as a major barrier to evidence synthesis [5,6].

### 3.2. Preparation Techniques and Their Downstream Biological Consequences

Most clinical PRP is produced via centrifugation-based separation of whole blood. Single-spin and double-spin approaches can yield different platelet enrichment and cellular contamination profiles. A comparative study found that double centrifugation can increase platelet concentration and yield while reducing red and white blood cell contamination, supporting its use for more “refined” PRP preparation in some settings [8]. However, the “best” method is not universal; preparation outcomes depend on the patient’s baseline platelet count and hematologic parameters. In a cohort of knee osteoarthritis patients, baseline platelet counts influenced fold-change enrichment in the final product, and leukocyte subtype distributions shifted during preparation (often toward lymphocyte predominance) [9]. This issue may be particularly relevant in urologic and sexual medicine populations, where diabetes, dyslipidemia, smoking, obesity, and aging are common. These factors can influence platelet function, inflammatory tone, and possibly the bioactive composition of the final PRP product. It is therefore plausible that identical processing steps may generate biologically non-identical PRP across different patient populations.

Activation is another variable with major implications. PRP may be injected non-activated, relying on tissue collagen/thrombin for in situ platelet activation, or activated ex vivo using calcium chloride or thrombin to trigger degranulation and growth factor release [7,10]. Activated vs. non-activated PRP likely differ in release kinetics, fibrin scaffold formation, and bioavailability of factors at the target site. Yet activation approaches are heterogeneous across studies, and mechanistic comparisons remain incomplete in the contexts of ED and pelvic tissue repair.

Storage and processing also matter. Cryo-processing has been explored to prolong the shelf life of platelet-rich autoplasma preparations, but evidence on whether growth factor content is preserved, diminished, or selectively altered is limited and protocol-dependent [11]. Newer strategies aim to manipulate not only platelets but also plasma growth factors, such as water-evaporation methods producing plasma rich in platelet- and plasma-derived growth factors that may improve in vitro cell viability [12]. Such innovations are promising for standardization, but they also widen the gap between “PRP” as a simple autologous concentrate and “platelet-derived biologics” as engineered products.

### 3.3. Beyond Platelets: Leukocytes, Plasma Proteins, and Omics Profiles

PRP is often simplistically described as a “platelet therapy,” but in reality, it is a composite biologic that may contain leukocytes, plasma proteins, cytokines, complement-related factors, lipid mediators, extracellular vesicles, and other molecular signals. A broad review of molecular actions of PRP highlights its mixed content and multi-pathway effects, including macrophage polarization and cytokine modulation [13]. The PRP “signaling secretome” is complex and includes pro-angiogenic and pro-fibrotic mediators, meaning that context and dose are likely decisive [14]. A striking implication is that PRP may not have a single directional effect (e.g., always anti-fibrotic); rather, it can push tissue responses depending on cellular composition, activation, and local microenvironment.

Multi-omics studies reinforce this heterogeneity. Metabolomic profiling demonstrates that even modest differences in preparation can transform metabolite signatures affecting inflammatory signaling, redox balance, energy metabolism, and activation pathways [15]. Proteomic analyses of platelet lysates likewise show that processing affects protein composition and potential functional properties [16]. For sexual medicine, where outcomes are sensitive to subtle neurovascular changes and placebo effects can be significant, these biochemical differences may translate into clinically meaningful variability, yet trials typically do not measure platelet dose, growth factor concentrations, or inflammatory cytokines in the final injectate.

Practical implications: for PRP to mature into an evidence-based intervention in urology and sexual medicine, “PRP characterization” should move from optional to routine: at minimum, platelet concentration, total platelet dose delivered, leukocyte profile (rich vs. poor), activation method, and red blood cell contamination should be reported for each trial and ideally linked to outcomes [5,6,13].

## 4. Mechanistic Rationale in Urology and Sexual Medicine

Mechanistic plausibility alone cannot justify clinical adoption, but it can inform (i) which patient phenotypes are most likely to respond, (ii) which formulations and dosing strategies are rational, and (iii) which endpoints best capture biological effects. Here, we summarize four interlocking mechanistic domains most relevant to urology and sexual medicine: nerve repair, angiogenesis/endothelial support, ECM remodeling/anti-fibrotic effects, and immune microenvironment optimization.

### 4.1. Nerve Regeneration: Neurotrophic Signaling and Schwann Cell Support

Peripheral nerve injury models provide the most direct evidence that platelet-derived products can influence axonal regrowth and remyelination. In a sciatic nerve injury model, PRP promoted peripheral nerve regeneration, with mechanistic signals implicating pathways relevant to repair and cell–matrix interactions [17]. In a distinct model using a PRP-loaded nerve guidance conduit for recurrent laryngeal nerve regeneration, PRP supported electrophysiologic recovery and histologic repair, highlighting the potential value of combining PRP with biomaterials to prolong factor delivery and provide structural guidance [18].

A plausible cellular mediator is the Schwann cell. Schwann cells are required for axonal guidance and myelination and respond to growth factors and ECM cues. In the nerve conduit study, PRP promoted Schwann-cell-associated signaling and improved markers of myelin repair, suggesting that PRP may work not only by acting on neurons but also by shaping glial and stromal components of the regenerative niche [18]. Translating to sexual medicine, cavernous nerve injury (e.g., after radical prostatectomy) triggers denervation, smooth muscle apoptosis, endothelial dysfunction, and fibrosis, pathways in which enhanced Schwann cell activity and remyelination could be meaningful.

Neurotrophic factors may also play a role. In a neuropathic orofacial pain model, PRP increased indicators linked to nerve regeneration, such as BDNF and Krox20, supporting the idea that PRP can induce a neurotrophic milieu [19]. More recently, PRP-derived exosomes were shown to “boost” mesenchymal stem cells (MSCs), enhancing their paracrine function and peripheral nerve regeneration potential via signaling pathways such as PI3K/Akt [20,21]. These data are conceptually important because they broaden PRP from a simple bolus of soluble growth factors to a biologic that can deliver extracellular vesicles and modulate recipient-cell behavior in a sustained fashion.

In urology-specific nerve injury, evidence is emerging. In a cavernous nerve injury model, PRP administration influenced axonal and collagen remodeling at the injury site, supporting a nerve-repair and anti-fibrotic hypothesis relevant to ED pathophysiology [22]. However, caution is required: animal models often use standardized injuries and tightly controlled timing, whereas human nerve injury varies in severity, chronicity, and comorbidity burden. It is therefore unlikely that a single PRP protocol will fit all neurogenic ED phenotypes.

### 4.2. Angiogenesis and Endothelial Support: VEGF and Beyond

Penile erection is fundamentally a hemodynamic event requiring intact endothelium, smooth muscle relaxation and adequate arterial inflow with veno-occlusive competence. PRP contains pro-angiogenic mediators, most notably VEGF, and can influence endothelial proliferation and migration [13,14]. In diabetic rat models of impaired tissue viability, activated PRP increased VEGF and angiogenesis, improving tissue survival in a flap model [23]. PRP-exosome work suggests additional mechanisms; for example, sphingosine-1-phosphate carried in PRP-derived exosomes promoted angiogenesis via S1PR1/AKT/FN1 signaling in diabetic wound healing [24]. While these are not penile models, diabetes is a major ED driver, and such mechanistic pathways could be relevant.

Yet the translation is not straightforward. ED is often an “end-organ” manifestation of systemic vascular disease. If endothelial dysfunction is driven by chronic cardiometabolic injury, the local angiogenic stimulus from PRP may be insufficient, or short-lived, unless systemic risk factors are addressed concurrently. This might partly explain why some ED trials observe early improvements that attenuate over time [25,26,27]. Additionally, PRP may differentially modulate angiogenesis depending on formulation and assay context: platelet-derived products can promote complex microvascular network formation in some 3D systems while producing variable results in simpler assays [28]. For ED trials, this implies that composition and activation state could meaningfully alter vascular outcomes, but such variables are rarely quantified.

### 4.3. ECM Remodeling and Fibrosis

Fibrosis within the corpora cavernosa (e.g., reduced smooth muscle-to-collagen ratio) is a central mechanism in many forms of ED and in Peyronie’s disease. PRP is often described as “anti-fibrotic,” but the biology is nuanced. In a scleroderma model, PRP combined with fat transplantation reduced fibrosis and increased angiogenic factor expression [29]. Conversely, some in vitro work suggests that PRP can both downregulate and upregulate pro-fibrotic genes depending on conditions, and the net effect on ECM remodeling may be context-dependent [30]. This is highly relevant to urology because PRP contains TGF-β, a mediator with well-known pro-fibrotic signaling; whether PRP’s overall effect is anti-fibrotic may depend on dose, timing, inflammatory milieu, and the balance of antagonistic mediators.

A practical interpretation is that PRP may be most useful in earlier, potentially reversible stages of tissue remodeling (mild-to-moderate ED, earlier fibrosis), while advanced structural disease may be less responsive. This matches the general pattern in regenerative medicine: once a tissue reaches a “point of no return” (extensive fibrosis, smooth muscle loss), growth factor supplementation alone may not restore normal architecture.

### 4.4. Immune Modulation and Microenvironment Optimization

PRP contains mediators that can modulate immune cell behavior, including macrophage polarization. In a sciatic nerve regeneration context, the autologous platelet-rich growth factor reduced M1 macrophages and modulated inflammatory microenvironments, supporting nerve repair [31]. In tissue engineering, PRP incorporated into conduits or hydrogels improved the regenerative microenvironment for peripheral nerve repair, consistent with sustained local immunomodulation and trophic signaling [32].

PRP also interacts with stem/progenitor cell biology. Multi-omics analysis showed that PRP promoted MSC proliferation, adhesion, and migration, with metabolic reprogramming toward anabolic processes [33]. Platelet-derived mitochondria transfer to MSCs has been reported as a novel mechanism that can enhance pro-angiogenic properties through metabolic effects [34]. These findings support combination strategies in which PRP serves as a “biologic adjuvant” to improve cell therapy performance, a concept that has already influenced orthopedics and may become increasingly relevant in urology.

However, immune modulation can also complicate the interpretation of symptoms and placebo response. For conditions with prominent pain components (e.g., bladder pain syndrome, vulvovaginal disorders), improvements may reflect changes in local inflammatory signaling rather than structural regeneration itself. Trials should therefore incorporate both symptom scores and objective biomarkers or imaging when feasible.

## 5. Current Clinical Applications of PRP in Urology and Sexual Medicine

### 5.1. PRP Treatment for Erectile Dysfunction

#### 5.1.1. Rationale for PRP in ED

ED is not a single disease entity; it is a final common pathway for vascular insufficiency, endothelial dysfunction, smooth muscle pathology, neurogenic injury, hormonal factors, and psychogenic contributors. The clinical promise of PRP rests on its capacity to modulate multiple tissue-level processes simultaneously (neurotrophic signaling, angiogenesis, immune polarization, ECM remodeling). However, the same multi-component nature that makes PRP attractive also makes it harder to test: if different ED phenotypes respond through different mechanisms, then trials that enroll heterogeneous populations without stratification may dilute true signals.

In most PRP-ED clinical studies, the target population has been men with mild-to-moderate ED, sometimes including vasculogenic or mixed etiologies, and less frequently, men with severe ED or major neurogenic injury. This matters because men with advanced cavernosal fibrosis or severe arterial disease may have limited capacity to respond to growth factor-based approaches, whereas men with earlier functional impairment might show measurable gains in erectile function scores and hemodynamic parameters.

A further complexity is that PRP composition varies across individuals. In men with ED, PRP growth factor concentrations can differ substantially, implying that the “dose” of bioactive mediators is not fixed even when the injected volume is standardized [35]. In principle, this variability could be exploited (personalized PRP characterization and dosing), but in practice, it more often becomes a confounder that contributes to inconsistent outcomes across trials.

#### 5.1.2. Outcomes Used in PRP-ED Trials

Most PRP-ED studies use validated patient-reported outcomes, particularly the erectile function domain of the International Index of Erectile Function (IIEF-EF) or the abbreviated IIEF-5 [27,36,37,38,39]. These outcomes are clinically relevant but can be sensitive to placebo effects, expectation, and behavioral factors (e.g., changes in sexual frequency, partner dynamics) over the trial period.

Objective measures are used less consistently. Some studies include penile duplex Doppler ultrasound metrics, such as peak systolic velocity (PSV) and end-diastolic velocity (EDV), to quantify arterial inflow and veno-occlusive function; others report intracavernosal pressure/mean arterial pressure ratios (ICP/MAP), mainly in animal models [4]. While Doppler parameters can strengthen mechanistic inference, they also depend on operator technique, pharmacologic stimulation protocols, and patient factors, and thus require strict standardization.

A practical implication for trial design is that ED PRP studies should ideally include (i) a validated sexual function score; (ii) at least one objective physiological endpoint (e.g., Doppler PSV) when feasible; and (iii) durability assessments beyond the early response window (e.g., 6–12 months), because short-term improvement does not necessarily indicate sustained tissue repair.

#### 5.1.3. Critical Synthesis of Randomized and Prospective Evidence in ED

Randomized and prospective clinical studies of intracavernosal PRP in ED suggest a possible efficacy signal, but that signal remains highly sensitive to study quality, comparator rigor, and adequacy of PRP characterization [25,26,36,38]. As summarized in Table 1, some placebo-controlled randomized trials reported higher rates of clinically meaningful improvement with PRP than with saline placebo, whereas others found no significant between-group superiority despite within-group improvement in both arms [25,36,38]. This inconsistency is not unexpected when considered alongside the substantial heterogeneity in enrolled populations, background therapies, injection schedules, and most importantly, the incomplete biologic description of the injected product [10,27,37,40].

A key interpretive pattern emerges when study design is considered together with efficacy results. The more rigorously controlled ED trials generally support at most a modest and still uncertain benefit, rather than a uniformly robust treatment effect. In contrast, single-arm studies and observational cohorts more often report favorable outcomes, including improvements in IIEF-based scores and, in some reports, penile Doppler parameters. However, these lower-level designs are inherently more vulnerable to placebo response, regression to the mean, sexual-behavior changes during follow-up, and confounding from concomitant therapies such as PDE5 inhibitors [27,37,40]. In practical terms, the apparent strength of the PRP signal tends to increase as internal validity decreases.

This contrast is central to the main argument of the present review. The current ED literature does not simply suffer from limited sample size; it suffers from biological ambiguity. In many studies, injected volume is reported, but platelet concentration, leukocyte content, activation strategy, erythrocyte contamination, and absolute platelet dose are not adequately described [5,6,25,27,36,38]. Consequently, even when a study is randomized, it often cannot determine whether a neutral or positive result reflects the true effect of PRP as a class, or merely the effect of one incompletely defined platelet-derived product [5,6,13]. This problem materially limits reproducibility and weakens the interpretability of both positive and negative trials.

Another recurring limitation is short follow-up [25,26,27,37,38]. This is particularly important for an intervention framed as regenerative. If PRP is expected to induce durable neurovascular recovery or structural remodeling, benefits should remain evident beyond the early post-treatment window. Short-term improvements in IIEF-EF or IIEF-5 alone cannot reliably distinguish true tissue repair from transient inflammatory modulation, behavioral adaptation, or expectancy effects [10,27,37,40]. The same concern applies to studies in which co-interventions, including PDE5i continuation or combination treatment strategies, complicate the attribution of benefit to PRP itself [42,43,44].

Accordingly, the current evidence supports a cautious but not dismissive conclusion: intracavernosal PRP may benefit a subset of men with mild-to-moderate ED, particularly those with less advanced disease, but the magnitude, durability, and phenotype specificity of that benefit remain uncertain [25,26,27,36,37,38,39,40,45,46]. At present, the most reproducible conclusion from the ED literature is not that PRP is definitively effective, but that study quality and reporting completeness materially shape the observed efficacy signal [5,6,27,37,40].

To facilitate interpretation of this evidence signal in relation to internal validity, a pragmatic mini risk-of-bias appraisal of the key ED studies is provided in Table 2.

#### 5.1.4. Aggregate Signal and Clinical Interpretation

Systematic reviews and meta-analyses generally report improvements in erectile function outcomes after PRP treatment, including pooled improvements in IIEF-based measures and, in some analyses, favorable comparisons versus placebo [27,37,44,47]. However, these syntheses necessarily inherit the same limitations as the primary studies: substantial heterogeneity in PRP formulation, platelet dose, leukocyte content, activation strategy, injection schedule, patient phenotype, co-interventions, endpoint definitions, and follow-up duration [10,27,37,46]. As a result, pooled estimates should be interpreted as evidence of a possible class-level signal under heterogeneous conditions, rather than proof of a standardized and reproducible therapeutic effect.

Importantly, the aggregate signal appears stronger when lower-quality or uncontrolled studies are included, and weaker when emphasis is placed on sham- or placebo-controlled randomized evidence [25,26,27,37,46]. This contrast is highly informative. It suggests that deficiencies in blinding, comparator rigor, protocol standardization, and PRP characterization may inflate the apparent efficacy of PRP in the broader literature [5,6,27,37]. Conversely, the neutral findings of better-controlled trials do not necessarily invalidate PRP as a biologic concept; rather, they indicate that the field still lacks a sufficiently standardized intervention to test rigorously and compare meaningfully across studies [5,6,13,27].

A further interpretive issue is that statistical improvement does not automatically translate into clinically meaningful benefit. In ED studies, responder analysis and minimal clinically important difference (MCID) thresholds are more informative than mean score changes alone, particularly because MCID depends partly on baseline severity [38]. Yet many studies and pooled analyses remain limited in their ability to provide severity-stratified responder interpretation [27,37,46]. This makes it difficult to determine whether observed improvements represent patient-perceived restoration of sexual function or only modest numerical change.

From a clinical perspective, PRP for ED should therefore be regarded as investigational or, at most, adjunctive, rather than guideline-established therapy [1,27,44,45,46,47,48]. If offered in clinical practice or research settings, it should be accompanied by explicit informed consent regarding the uncertainty of benefit, careful documentation of PRP preparation and delivery variables, and prospective outcome tracking using validated measures and, where feasible, objective physiologic endpoints such as penile duplex Doppler ultrasound [25,27,37,38]. The main priority for the field is no longer simply to accumulate more PRP studies, but to generate better-defined, biologically interpretable, sham-controlled trials capable of identifying which PRP formulations, treatment schedules, and ED phenotypes are most likely to derive meaningful benefit [5,6,27,37,46].

### 5.2. Neurogenic ED and the Place of PRP Within a Broader Regenerative Landscape

#### 5.2.1. Pathophysiology

Radical prostatectomy and other pelvic surgeries can injure the cavernous nerves, initiating a cascade of denervation-associated changes: smooth muscle apoptosis, endothelial dysfunction, hypoxia, and cavernosal fibrosis. These processes can convert potentially reversible dysfunction into more refractory ED over time, even when nerve-sparing techniques are used [49,50]. Conventional penile rehabilitation approaches (e.g., PDE5i) target hemodynamics but may not fully address neural repair and microenvironment remodeling.

#### 5.2.2. Regenerative Strategies Beyond PRP: Stem Cells, Biomaterials, and Molecular Targets

Preclinical regenerative strategies include stem cell therapies, biomaterial-assisted delivery, and targeted molecular interventions. Human placental stem cells and umbilical cord-derived MSCs have shown neurovascular and smooth muscle regenerative effects in rodent models of pelvic neurovascular injury and cavernous nerve injury, with improvements in functional and histologic outcomes [51,52]. Biomaterial approaches aim to improve cell retention and paracrine delivery; for instance, adipose-derived stem cells embedded in an erythropoietin-loaded hydrogel enhanced nerve regeneration and penile tissue preservation in bilateral cavernous nerve injury models [53]. Nanofiber scaffolds seeded with stem cells have also been explored to improve neural differentiation and corporal tissue architecture after nerve injury [54].

Molecular strategies include agents targeting Wnt signaling or neurotrophin pathways. A flavonoid derivative (YS-10) improved erectile parameters and reduced fibrosis and endothelial/neural dysfunction in a cavernous nerve injury model, potentially via Wnt pathway activation [55]. Neutralizing antibody strategies against proNGF have been reported to preserve cavernous neurovasculature and rescue erectile function by altering neurotrophic and angiogenic factor expression [50].

These approaches illustrate two important points for PRP positioning: (i) neurogenic ED may require multi-target interventions because nerve injury drives downstream vascular and fibrotic pathology; and (ii) PRP may act best as a component of multimodal rehabilitation (e.g., paired with hemodynamic support or with other regenerative stimuli), rather than as a stand-alone solution in severe nerve injury.

#### 5.2.3. PRP for Neurogenic ED: What We Can Infer from Preclinical Evidence

While direct clinical evidence for PRP, specifically in post-prostatectomy neurogenic ED, remains limited in the provided reference set, preclinical data support plausibility. In rodent models, PRP has been linked to preservation of erectile function after bilateral cavernous nerve injury, with mechanistic emphasis on cytokines such as CXCL5 [56]. In diabetic rats, intracavernosal PRP improved erectile function and reduced mortality, suggesting broader systemic interactions or improvements in metabolic stress responses [57]. Animal work also suggests that PRP may enhance axonal regeneration and limit collagen deposition in cavernous nerve injury contexts [22].

However, translating these findings to the clinic requires caution for at least three reasons:

(1) Timing and dosing: animal studies often administer PRP early after injury; many human patients present later with established fibrosis.
(2) Injury severity: surgical traction, thermal injury, and patient-specific anatomy create heterogeneous nerve damage patterns.
(3) Comorbidities: diabetes, vascular disease, and aging can impair repair capacity and platelet function, potentially reducing PRP potency.

These findings justify continued investigation, but direct clinical evidence for PRP specifically in post-prostatectomy neurogenic ED remains limited.

### 5.3. Combination Therapy Strategies: PRP with Li-ESWT, PDE5is, and Other Modalities

Combination strategies are biologically appealing because PRP may not act through a single dominant mechanism. Pairing PRP with therapies that improve hemodynamics, induce endogenous repair signaling, or stabilize the tissue microenvironment may be more effective than monotherapy in selected patients.

#### 5.3.1. PRP Plus Li-ESWT

Low-intensity extracorporeal shockwave therapy (Li-ESWT) is a non-invasive regenerative modality proposed to stimulate angiogenesis and recruit endogenous repair pathways in ED. Reviews of Li-ESWT after prostate cancer treatment suggest mixed results and emphasize protocol variability and limited long-term data [58]. Mechanistically, Li-ESWT may “prime” tissue by inducing controlled microtrauma and pro-angiogenic signaling, potentially enhancing responsiveness to growth factors.

Combination therapy with PRP has been explored clinically in ED, with some studies reporting superior outcomes compared with either modality alone. A prospective comparative study reported that combining shockwave therapy with PRP produced larger and more sustained improvements in IIEF-5 and PSV in arteriogenic ED than monotherapy [59]. A systematic review and meta-analysis also evaluated PRP alone or combined with Li-ESWT in ED and suggested benefit, while acknowledging limitations in trial heterogeneity and quality [44]. Importantly, these combination studies often remain observational or have design limitations, and their protocols vary, making it difficult to generalize.

Although promising, these data remain insufficient to establish synergy conclusively. The field urgently needs standardized, sham-controlled factorial trials that can separate the individual and interactive effects of PRP and Li-ESWT.

#### 5.3.2. PRP Plus Conventional Pharmacotherapy (e.g., PDE5i)

A pragmatic and potentially scalable strategy is combining PRP with PDE5i, because it links symptomatic hemodynamic support with a putative biologic repair strategy. In a diabetic patient with ED not responding to on-demand PDE5i, adding intracavernosal PRP to daily tadalafil improved erectile function and erection hardness scores, suggesting possible synergy or rescue of PDE5i responsiveness [42]. While encouraging, this evidence should be interpreted in the context of potential confounding (behavioral changes, regression to the mean, and improved glycemic control). Nevertheless, the study supports a broader principle: regenerative therapies may perform better when the functional environment (oxygenation, hemodynamics) is optimized, which could help sustain newly formed vessels and prevent fibrosis progression.

#### 5.3.3. Implications for Trial Design

The key problem with the current combination therapy literature is not a lack of rationale, but a lack of clean causal separation. Most studies cannot determine whether the observed benefit reflects PRP, the co-intervention, or their interaction. Future studies should therefore use factorial or otherwise appropriately controlled designs to test additive and interactive effects directly rather than assuming synergy.

## 6. Other Applications Across Urology and Sexual Medicine

Evidence for PRP beyond ED is expanding, but the overall pattern is similar: strong biological plausibility, frequent early positive signals, and a shortage of standardized protocols and high-quality controlled trials. Because these conditions differ in target tissue, delivery route, and endpoints, PRP should be considered an indication-specific biologic rather than a generic intervention, whose relevance depends on tissue target, disease phase, and delivery route.

### 6.1. Peyronie’s Disease (PD)

PD is characterized by localized fibrosis of the tunica albuginea with plaque formation, penile curvature, pain (often in the early phase), and frequently concomitant ED. The mechanistic rationale for PRP in PD is straightforward: PRP contains mediators that can influence inflammation resolution, angiogenesis, and ECM remodeling, and it may modulate fibrotic signaling pathways. Nonetheless, the clinical evidence base remains limited relative to the speed of clinical uptake.

A 2024 systematic review evaluated PRP in both ED and PD and highlighted that the PD literature is heterogeneous and constrained by study design limitations [27]. A 2025 narrative review of intralesional and topical PD treatments situates PRP among experimental approaches, reflecting that guideline-level endorsement remains premature [46]. More recently, a large prospective cohort reported that intraplaque PRP injections were associated with rapid reductions in curvature and improvements in sexual function scores [45]. While such observational data are encouraging, they cannot fully account for natural history, regression to the mean, co-interventions, and expectation effects, particularly in PD, where pain and curvature may fluctuate.

Combination strategies are also reported. Low-intensity extracorporeal shockwave therapy (Li-ESWT) plus PRP has been described as an effective combination for chronic-phase PD in some clinical studies, with improvements in curvature and patient satisfaction and few adverse events [60]. Similar reports exist in acute-phase PD with reductions in plaque size and symptom measures [61,62]. These findings should be interpreted cautiously: phase-specific PD outcomes can improve spontaneously, and controlled trials with standardized plaque imaging, curvature measurement protocols, and long-term follow-up are still needed. Table 3 illustrates the structured evidence of PRP for PD.

Critical synthesis

Taken together, the PD literature suggests a preliminary symptom and curvature signal, but the evidence remains substantially less mature than the ED literature. Positive findings arise mainly from observational studies or multimodal protocols in which the independent effect of PRP cannot be isolated confidently. The limited randomized evidence does not yet establish a consistent efficacy pattern. Therefore, the main conclusion is not that PRP is proven for PD, but that current studies are too heterogeneous in disease phase, co-interventions, and injectate characterization to determine whether a specific PRP approach confers reproducible benefit.

### 6.2. Interstitial Cystitis/Bladder Pain Syndrome (IC/BPS)

Lower urinary tract conditions such as IC/BPS are challenging because symptom severity is high, treatment responses are variable, and placebo responses can be substantial. PRP has been explored as an intravesical or submucosal injection therapy intended to modulate inflammation and improve urothelial integrity repair.

One study suggested that tumor necrosis factor-α (TNF-α) levels in PRP might be associated with treatment outcomes in patients with IC/BPS or recurrent urinary tract infection, raising the possibility of biomarker-guided PRP selection [63]. In addition, intravesical PRP injection has been reported with an emphasis on efficacy and safety, but broader conclusions are limited by the need for more controlled studies and standardized protocols [64]. A standardized protocol for modified PRP collection for IC/BPS has also been reported, reflecting a growing recognition that reproducibility and product definition are prerequisites for meaningful trials [65]. These findings remain preliminary and require confirmation in rigorously controlled trials using symptom scores alongside objective measures such as urinary biomarkers, cystoscopic features, or validated phenotyping frameworks. Table 4 illustrates structured evidence of PRP for IC/BPS.

Critical synthesis

The IC/BPS evidence is best regarded as proof-of-concept rather than practice-changing. Early studies suggest that submucosal or intravesical PRP may improve symptoms in selected refractory patients, but the absence of robust controls and the high placebo sensitivity of this condition sharply limit causal inference. Importantly, IC/BPS may be one of the most informative settings for biomarker-linked PRP research, because both disease heterogeneity and injectate heterogeneity are likely to influence response. Until controlled studies integrate symptom outcomes with biologic and urothelial markers, the clinical role of PRP in IC/BPS should remain investigational.

### 6.3. Female Sexual Medicine and Pelvic Floor Disorders

PRP use in female sexual medicine has increased, often driven by unmet needs in hormone-contraindicated populations (e.g., breast cancer survivors) and by interest in minimally invasive tissue restorative approaches. However, this field also faces the same central problem: protocols vary, and controlled evidence is limited.

#### 6.3.1. Genitourinary Syndrome of Menopause (GSM)

A clinical study evaluated PRP for GSM in breast cancer survivors and reported outcomes relevant to vulvovaginal symptoms and quality of life [66]. A randomized trial compared intravaginal PRP therapy versus local hormonal treatments and assessed sexual quality-of-life outcomes in postmenopausal women, supporting potential efficacy while underscoring the need for replication and standardized endpoints [67]. These studies are important because they address a clinically constrained population and because GSM outcomes can be measured via both subjective and objective indices (vaginal health index, symptom scales).

#### 6.3.2. Stress Urinary Incontinence (SUI)

Preclinical evidence suggests that PRP may improve neuromuscular and proprioceptive abnormalities in postpartum SUI models, with improvements in leak point pressures and muscle spindle morphology [68]. Clinically, a pilot study reported that local autologous PRP injection improved SUI symptoms without significant adverse effects [69]. Urethral sphincter PRP injections for intrinsic sphincter deficiency have also been explored in proof-of-concept clinical work, reflecting interest in targeted regenerative approaches for continence mechanisms [70]. Other prospective studies and sham-controlled randomized trials demonstrated benefit with a repeat periurethral injection protocol, showing improvement in both patient-reported symptoms and pad-test outcomes [71,72], whereas another placebo-controlled randomized trial using a single mid-urethral anterior vaginal wall injection failed to show superiority over saline [73]. All studies suggested an acceptable short-term safety profile, with no serious treatment-related adverse events reported. These discordant findings indicate that any potential benefit of PRP may be protocol-dependent and highlight the need for standardized PRP characterization, optimized dosing schedules, and adequately powered confirmatory trials. Table 5 illustrates structured evidence of PRP for SUI.

#### 6.3.3. Pelvic Organ Prolapse (POP)

A review addressing PRP products in urogynecological disorders describes emerging roles, including tissue remodeling and collagen-related changes, but high-quality comparative evidence remains sparse [74]. As with other indications, the key methodological challenge is distinguishing true tissue reinforcement from nonspecific symptom fluctuations and confounding by concurrent pelvic floor therapy or surgical interventions.

#### 6.3.4. Vulvovaginal Disorders and Lichen Sclerosus

PRP has been used in vulvovaginal disorders and lichen sclerosus, often with symptomatic improvement reported, but variable protocols and limited controlled data. A systematic review in vulvovaginal disorders reflects both interest and evidence constraints [75]. PRP for lichen sclerosus has been discussed in the literature, but the overall evidence remains limited and heterogeneous [76]. Notably, a comprehensive review of genital lichen sclerosus highlights PRP and adipose-derived stem cell therapy as regenerative approaches of interest, again emphasizing the need for standardized protocols and rigorous clinical evaluation [77].

A broader systematic review of PRP injections for female sexual dysfunction and SUI concluded that PRP appears generally safe and may improve symptoms, but the level of evidence is limited by heterogeneous study designs and reporting [78]. This aligns with the broader theme of the field.

Critical synthesis

Across female sexual medicine and pelvic floor disorders, the overall evidence pattern mirrors that seen in male sexual medicine: encouraging early signals, acceptable short-term tolerability, but limited standardization and insufficient comparative rigor. The most clinically relevant question is not whether PRP can produce short-term symptom improvement, but whether a clearly defined formulation delivered to a specific tissue target produces durable and objectively measurable functional benefit. At present, that question remains incompletely answered.

### 6.4. Male Infertility and Reproductive Applications: Mostly Early-Stage Evidence

PRP has been investigated as a supportive scaffold or trophic supplement for spermatogonial stem cell (SSC) culture and self-renewal in vitro, suggesting potential translational relevance to fertility preservation and SSC expansion strategies [79]. These findings are useful for mechanistic hypothesis generation, but they do not yet define a safe, standardized, or clinically validated andrological application. This area should therefore be framed as exploratory and preclinical.

## 7. Discussion

### 7.1. What This Review Adds

The major challenge in interpreting the PRP literature in urology and sexual medicine is not the absence of mechanistic plausibility. On the contrary, preclinical evidence consistently suggests that platelet-derived biologics may influence nerve repair, angiogenesis, immune signaling, and matrix remodeling. The principal problem is that clinical studies often evaluate undercharacterized and biologically non-equivalent products under the same label. As a result, the field risks asking the wrong question. Rather than asking whether “PRP works” as a single intervention, a more appropriate framework is to ask: which PRP formulation, delivered how, in which phenotype, at what disease stage, and with what clinically meaningful endpoint?

This review contributes a cross-indication translational perspective that integrates mechanistic rationale, trial methodology, and implementation barriers across multiple urologic and sexual medicine conditions. Most importantly, it proposes a practical reporting framework intended to reduce one of the field’s most remediable weaknesses: inadequate description of the PRP product and protocol.

### 7.2. Safety and Adverse Events

Across current urology and sexual medicine applications, PRP is generally described as safe, largely because it is autologous and therefore has minimal immunogenic risk. Systematic reviews focused on ED report favorable short-term safety profiles, with no consistent signals of serious adverse events [38]. Mild local reactions, such as pain, swelling, and hematoma, are the most commonly reported issues in injection-based protocols, usually self-limited [36,38]. Rare complications such as fibrotic plaque formation have been reported but appear uncommon in available series [41].

In female sexual medicine and SUI, PRP injection protocols (often multiple sessions, 2–6 mL monthly over several months) are generally well tolerated in the published literature, with mostly mild local discomfort and limited serious adverse event reporting [78]. Importantly, “absence of evidence” is not “evidence of absence”: many studies are underpowered for safety, follow-up is often short (commonly up to ~6 months), and adverse event definitions are not always standardized.

Key safety gaps include:

(1) Long-term safety of repeated injections, especially when PRP is administered in multiple courses or combined with other regenerative modalities.
(2) Product variability and contamination risks related to preparation protocols and sterility in real-world settings.
(3) Risk in comorbid populations, such as diabetes or vascular disease, where baseline platelet function and inflammatory status may differ and where local tissue perfusion may be compromised.

Practical recommendation: Studies should implement standardized adverse event reporting (local vs. systemic, severity grading, timing), include longer follow-up where feasible, and document co-interventions (PDE5i use, Li-ESWT, lifestyle changes), which can confound both efficacy and safety interpretation.

### 7.3. Ethical and Regulatory Considerations

The regulatory classification of PRP varies widely across jurisdictions. In some settings, it is treated as a minimally manipulated autologous product with relatively permissive oversight; in others, it is subject to stricter biologic-like regulation. A narrative review of PRP regulations in South America illustrates this variability, ranging from structured governance to restrictions limiting use to experimental contexts [80]. Such heterogeneity influences not only clinical availability but also the quality of PRP preparation, standardization, and reporting.

Ethically, the central risk is that clinical enthusiasm and market promotion exceed the strength of evidence, especially in sexual medicine, where patient vulnerability, stigma, and high willingness to try novel interventions can be exploited. Scholarly reviews in sexual medicine have emphasized both the promise and the need for caution with PRP and cellular therapies, particularly given the limited number of high-quality trials and the variability in protocols [42,45]. Informed consent must explicitly communicate (i) investigational status for many indications, (ii) uncertainty regarding magnitude and durability of benefit, (iii) variability in PRP preparations across clinics, (iv) alternative evidence-based therapies, and (v) cost considerations and opportunity costs.

Transparent consent is especially important in ED, PD, and IC/BPS, where placebo effects and subjective endpoints are substantial and where repeated treatment sessions can impose financial and psychological burdens.

### 7.4. Why Studies Disagree: A Critical Synthesis of Heterogeneity

The most important barrier to evidence-based integration of PRP into urology and sexual medicine is not a lack of biological plausibility. On the contrary, preclinical and translational data consistently suggest regenerative, angiogenic, and neurotrophic potential. Rather, the persistent inconsistency in clinical outcomes largely reflects profound heterogeneity-both in the PRP product itself and in the way it is delivered in clinical settings. Without addressing this variability, attempts to synthesize evidence or formulate standardized recommendations remain inherently constrained.

#### 7.4.1. PRP Product Heterogeneity

PRP products differ in (i) platelet concentration and total platelet dose delivered, (ii) leukocyte content (leukocyte-rich vs. leukocyte-poor), (iii) red blood cell contamination, (iv) activation method (activated vs. non-activated), (v) preparation device and centrifugation protocol, and (vi) storage/processing conditions.

Scoping and systematic reviews in other specialties show that many PRP investigations do not report full protocols and composition, limiting reproducibility and cross-study comparison [5,6]. This is equally true in sexual medicine, and it undermines meta-analysis interpretability: pooled results may combine biologically distinct products under a single label.

Physiological factors also contribute. PRP variability is affected by age, baseline platelet count, and systemic factors; growth factor concentrations can vary among individuals [35,81,82]. This variability is likely amplified in ED populations, where cardiometabolic disease and smoking are common and may alter platelet function and inflammatory milieu.

#### 7.4.2. Protocol Heterogeneity

Beyond the variability in the PRP product itself, substantial inconsistency exists in treatment protocols across urological and sexual medicine studies, particularly in erectile dysfunction (ED) trials.

Injection volumes per session vary, often ranging from a few milliliters to larger volumes without a clear biological justification. The number of treatment sessions and the intervals between them differ considerably, with protocols employing weekly, biweekly, or monthly schedules. Few studies provide a mechanistic rationale for these regimens, and most lack pharmacodynamic modeling to guide optimal frequency.

The route and anatomical target of delivery also vary. While intracavernosal injection is the most common approach in ED studies, injection techniques, anatomical landmarks, and use of imaging guidance are inconsistently described. The absence of standardized injection mapping raises the possibility that variability in tissue distribution contributes to differences in response.

Moreover, PRP is frequently administered alongside co-interventions such as PDE5i, Li-ESWT, or structured lifestyle modification. Although such combinations may reflect real-world practice, they confound the attribution of clinical benefit to PRP alone. In many trials, the relative contribution of adjunctive therapies remains unclear.

Taken together, this diversity in dose, frequency, route, and concomitant treatments makes it difficult to establish reproducible treatment algorithms. Importantly, when patients fail to respond, it becomes challenging to determine whether non-response reflects true biological refractoriness or simply suboptimal product composition and delivery parameters.

### 7.5. Endpoint Heterogeneity and Follow-Up Duration

Studies use different primary outcomes (IIEF-EF, IIEF-5, EHS, Doppler PSV/EDV, satisfaction scores), different definitions of response, and different follow-up durations. Without harmonized endpoints, trials cannot be compared reliably, and meta-analyses are forced to pool partially incompatible measures.

Short follow-up is particularly problematic for regenerative claims. True structural remodeling and nerve repair should manifest as durable improvements; transient benefits may reflect short-lived anti-inflammatory effects or placebo response.

### 7.6. Patient Selection and Disease Stage

ED etiologies (vasculogenic, neurogenic, mixed) likely respond differently to PRP. Severe neurogenic injury and advanced fibrosis may be less amenable to PRP monotherapy, whereas mild-to-moderate vasculogenic ED may show stronger responses. Trials that do not stratify by etiology and baseline severity risk reporting averaged effects that obscure meaningful subgroup differences.

Diabetes deserves special attention. Platelet dysfunction and altered growth factor release in diabetes may reduce PRP efficacy, and the broader literature has even explored allogeneic PRP approaches in diabetic foot contexts as a workaround when autologous platelet function is compromised [83]. While this is beyond sexual medicine, it highlights a mechanistically plausible concern: autologous PRP may not be equally potent across comorbidity groups.

### 7.7. Recommendations for Future Trials and Standardization

To move PRP from “promising but inconsistent” to evidence-based practice, future urology and sexual medicine studies should adopt a minimum methodological standard comparable to drug trials.

#### 7.7.1. The Minimum PRP Reporting Checklist for Urology/Sexual Medicine Trials (PRP-Uro Checklist)

To move the field from promising but inconsistent evidence toward reproducible science, we propose a Minimum PRP Reporting Checklist for Urology/Sexual Medicine Trials (Minimum PRP-Uro Checklist) (Table 6):

#### 7.7.2. Genesis and Justification of the PRP-Uro Checklist

The PRP-Uro Checklist was developed as an indication-focused adaptation of recurring reporting domains identified across the PRP literature, rather than as a de novo framework created in isolation. Existing classification and reporting systems in PRP research, such as those emphasizing platelet concentration/dose, leukocyte content, activation status, erythrocyte contamination, and preparation method, have highlighted the need for biologic characterization across specialties. However, these frameworks are not tailored to the specific interpretive needs of urology and sexual medicine, where treatment effects are often influenced by disease phenotype, anatomical target, injection mapping, co-interventions, placebo sensitivity, and the availability of both subjective and objective functional endpoints.

Accordingly, the PRP-Uro Checklist was designed by integrating two evidence streams: (i) general PRP reporting principles repeatedly emphasized in the broader regenerative medicine literature; and (ii) the specific deficiencies repeatedly identified during this review across ED, PD, SUI, IC/BPS, and female sexual medicine studies. The resulting checklist therefore aims not only to characterize the injected biologic, but also to improve the interpretability of indication-specific delivery, outcome assessment, and safety reporting.

The checklist is intended as a practical minimum reporting tool rather than a definitive consensus standard. Future refinement could proceed through formal multidisciplinary consensus methods involving experts in urology, andrology, urogynecology, regenerative medicine, transfusion medicine, trial methodology, and journal editing. Validation could include (i) content validation by expert panel review; (ii) pilot application to a representative sample of published PRP studies to assess completeness and usability; (iii) inter-rater reliability testing among independent reviewers; and (iv) prospective use in protocol development to determine whether it improves reporting completeness and cross-study comparability.

#### 7.7.3. How the Checklist Can Be Used in Practice

The PRP-Uro Checklist is intended not only as a reporting aid for investigators but also as a practical appraisal tool.

(i) Authors can use it prospectively during protocol development to ensure that the biologic intervention is defined before recruitment begins.
(ii) Peer reviewers can use it to determine whether a manuscript provides enough detail to support reproducibility and biological interpretation.
(iii) Editors and journals can adopt it as a supplementary reporting requirement for PRP-based interventional studies in urology and sexual medicine.
(iv) Professional societies and collaborative groups may use it as a starting point for broader consensus-building.

As a future dissemination strategy, the checklist could be refined through multidisciplinary consensus methods and aligned with wider reporting-guideline ecosystems.

#### 7.7.4. Illustrative Application of the Checklist

The checklist also helps explain why some published studies are difficult to interpret. A trial reporting only “5 mL intracavernosal PRP administered twice” without platelet concentration, leukocyte status, activation method, or handling conditions provides procedural information but not enough biological detail to support replication. By contrast, a trial that reports baseline platelet count, final concentration, estimated platelet dose, leukocyte profile, activation protocol, preparation system, co-intervention rules, and standardized adverse event collection becomes interpretable even if its efficacy result is neutral. Better reporting does not guarantee a positive study, but it does guarantee a more scientifically useful one.

#### 7.7.5. Trial Design Priorities

(1) Use double-blind, placebo/sham-controlled designs when feasible [27,38].
(2) Formal assessment of blinding integrity in injection-based studies.
(3) Biologically meaningful characterization of the PRP product.
(4) Prespecified severity-stratified responder definitions and MCID thresholds.
(5) Objective endpoints alongside symptom measures where feasible.
(6) Longer follow-up, ideally at least 6–12 months for ED and other regenerative claims.
(7) Explicit control and reporting of co-interventions.
(8) Phenotype-driven enrollment or stratification by etiology, severity, and comorbidity. [43].

## 8. Conclusions

PRP is a biologically plausible autologous therapy with mechanisms relevant to urology and sexual medicine, including neurotrophic support, angiogenesis modulation, immune polarization, and ECM remodeling. In ED, controlled trials and meta-analyses suggest potential improvements in erectile function in selected populations, but results are inconsistent and durability is incompletely established. Beyond ED, early studies in PD, IC/BPS, SUI, and selected female sexual medicine indications are promising but remain constrained by heterogeneous protocols and limited high-quality comparative evidence.

The central obstacle to evidence-based adoption is not a lack of interest, but a lack of standardization. PRP is too often treated as a uniform intervention despite substantial variation in composition, processing, and delivery. Advancing the field will require stricter product definition, indication-specific biologic rationale, standardized reporting, severity-aware clinical interpretation, and rigorously designed controlled trials with longer follow-up. Until these conditions are met, PRP should generally be positioned as investigational or adjunctive in most urological and sexual medicine indications, with transparent patient counseling and systematic outcome tracking. To facilitate more reproducible and clinically interpretable research, we propose the PRP-Uro Checklist as a practical minimum reporting framework for future studies.

## Figures and Tables

**Table 1:** Structured evidence table of PRP for erectile dysfunction.

Study	Population	Design/Sample Size	PRP Preparation/Product Characterization	Delivery Protocol/Comparator	Main Efficacy Findings	Safety Findings	Strengths	Limitations
Poulios et al., 2021 [38]	Men with mild-to-moderate vasculogenic ED	Double-blind RCT; *n* = 60	Magellan Autologous Platelet Separator; PRP composition incompletely characterized in terms of leukocyte content, growth factors, and absolute platelet dose	Intracavernosal PRP; 2 sessions, 1 month apart; placebo saline comparator	MCID achieved in 76% vs. 25% at 1 month, 69% vs. 39% at 3 months, and 69% vs. 27% at 6 months in PRP vs. placebo	No major adverse events; no hemorrhagic adverse events reported	Best-described sham-controlled ED RCT; clinically interpretable responder analysis	Small sample size; per-protocol denominator changes; incomplete PRP biologic characterization
Masterson et al., 2023 [25]	Men aged 30–75 with organic ED, IIEF 11–25	Prospective double-blind placebo-controlled RCT; *n* = 61	Arthrex Angel system; 120 mL blood processed to ~5 mL PRP; platelet/leukocyte characterization incomplete in publication summary	2.5 mL per corpus cavernosum at each of 2 sessions, 28 ± 7 days apart; saline comparator	MCID at 1 month after second injection: 58.3% vs. 53.6%, *p* = 0.7; no significant between-group superiority despite within-group improvements in both arms	One new plaque in PRP arm; one hematoma in placebo arm; no major complications	Well-controlled design; careful placebo comparator; transparent neutral result	Underpowered; attrition; substantial placebo response; incomplete biologic dosing detail
Shaher et al., 2023 [36]	Sexually active men with mild-to-moderate vasculogenic ED	Randomized double-blind placebo-controlled study; *n* ≈ 100 analyzed as 50/50 in systematic review table	Autologous PRP; preparation details incompletely reported	3 mL into each corpus cavernosum at 3 sites; repeated twice at 2-week intervals; saline comparator	MCID at 1, 3, and 6 months favored PRP: 76% vs. 18%, 72% vs. 16%, 70% vs. 16%	No major complications reported	Larger sample than most ED PRP trials	Blinding/randomization details insufficiently reported; incomplete between-group continuous outcome reporting; no severe ED
Ragheb et al., 2024 [26]	Men with mild-to-moderate ED	Prospective randomized comparative study; *n* = 52	PRFM rather than liquid PRP; Magellan system with CaCl_2_ activation	Intracavernosal PRFM, 3 injections; saline comparator	No significant between-group difference in IIEF outcomes	No serious complications reported	Randomized comparative design	Product was PRFM, limiting comparability with the PRP literature; small sample; incomplete product characterization
Francomano et al., 2025 [39]	Vasculogenic ED, PDE5i non-responders	Prospective single-arm; *n* = 150	5 mL PRP after 1500 rpm × 15 min; composition incompletely characterized	Single intracavernosal session, 5 mL per corpus cavernosum	IIEF-5 improved from 12 ± 2.6 to 19 ± 3.0; PSV improved from 32 ± 5.5 to 42 ± 7.6 cm/s	Dull pain in 16; slight hematoma in 2	Largest prospective ED PRP cohort	No control; short follow-up; single-arm design
Taş et al., 2021 [41]	Treatment-naïve vasculogenic ED with metabolic syndrome	Prospective single-arm; *n* = 31	PRP concentration reported as 1000–2000 × 10^3^/µL	3 injections, 15 days apart; 3 mL into each corpora cavernosa with penile clamp	61.3% improved; IIEF-EF significantly improved over 6 months	Mild bruising; one 4 mm ventral fibrotic plaque	Some platelet concentration reporting	No control group; small sample
Zaghloul et al., 2021/2022 [42,43]	PDE5i non-responders; one study focused on diabetic vs. non-diabetic men	Prospective cohorts	Double-spin preparation; composition incompletely reported	Repeated intracavernosal PRP plus high-dose PDE5i continuation	Significant IIEF-5 improvement reported; one study showed duplex parameter improvement	Mild injection pain; no major events	Suggests feasibility in difficult populations	Major confounding from concurrent tadalafil and on-demand vardenafil; no controls

Abbreviations: ED, erectile dysfunction; IIEF, International Index of Erectile Function; IIEF-EF, erectile function domain; MCID, minimal clinically important difference; PDE5i, phosphodiesterase type 5 inhibitor; PRFM, platelet-rich fibrin matrix; PRP, platelet-rich plasma; PSV, peak systolic velocity.

**Table 2:** Mini risk-of-bias table for studies of PRP in erectile dysfunction.

Study	Design	Selection Bias	Performance/Detection Bias	Intervention Reporting Bias	Attrition/Reporting Bias	Overall Risk of Bias
Poulios et al., 2021 [38]	Double-blind RCT	Some concerns: randomized, but allocation concealment details not clearly reported in the evidence table	Low risk: double-blind, saline placebo comparator	High risk/some concerns: PRP composition incompletely characterized, including leukocyte content, growth factors, and absolute platelet dose	Some concerns: per-protocol denominator changes noted despite clinically interpretable responder analysis	Some concerns
Masterson et al., 2023 [25]	Double-blind placebo-controlled RCT	Some concerns: randomized design, but allocation concealment not fully detailed in the summary	Low risk: placebo-controlled, double-blind	Some concerns: device and blood volume reported, but platelet/leukocyte characterization and biologic dose incomplete	Some concerns: underpowered, with attrition; neutral result transparently reported	Some concerns
Shaher et al., 2023 [36]	Randomized double-blind placebo-controlled study	Some concerns to high risk: randomization process insufficiently reported	Some concerns: described as double-blind, but blinding procedures insufficiently detailed	High risk: preparation details incompletely reported	Some concerns to high risk: incomplete between-group continuous outcome reporting	High risk/some concerns
Ragheb et al., 2024 [26]	Prospective randomized comparative study	Some concerns: randomized, but allocation/concealment details unclear	Some concerns: comparator present, but blinding not clearly stated	High risk: PRFM rather than conventional liquid PRP; incomplete product characterization limits comparability	Some concerns: no significant between-group difference reported, but sample small and reporting depth limited	Some concerns to high risk
Francomano et al., 2025 [39]	Prospective single-arm study	High risk: no randomization or control group	High risk: no blinding; uncontrolled design	High risk: PRP composition incompletely characterized	High risk: single-arm pre–post design with short follow-up	High risk
Taş et al., 2021 [41]	Prospective single-arm study	High risk: no randomization or control group	High risk: no blinding; uncontrolled design	Some concerns: platelet concentration reported, but broader biologic characterization incomplete	High risk: small sample, no control group	High risk
Zaghloul et al., 2021/2022 [42,43]	Prospective cohort studies	High risk: no randomization or control group	High risk: no blinding; strong confounding from concomitant PDE5i continuation	Some concerns to high risk: composition incompletely reported	High risk: major confounding and uncontrolled observational design	High risk

This mini risk-of-bias table provides a pragmatic study-level appraisal based on the structured evidence table rather than a formal RoB 2 or ROBINS-I assessment. Across the ED literature, the main recurring concerns were incomplete reporting of allocation methods, limited PRP biologic characterization, small sample size, attrition, and in non-randomized studies, absence of a control group with substantial risk of confounding from concomitant therapies. Although labeled here as a “mini risk-of-bias” summary for the ED evidence base, only four studies were randomized comparative trials; the remaining studies are included to provide a complete overview of internal validity.

**Table 3:** Structured evidence table of PRP for Peyronie’s disease.

Study	Population/Phase	Design/Sample Size	PRP Preparation/Characterization	Delivery Protocol/Comparator	Main Efficacy Findings	Safety Findings	Strengths	Limitations
Dachille et al., 2025 [45]	PD patients; phase not explicitly stated	Prospective single-arm large cohort; *n* = 72	Intraplaque PRP; composition not fully detailed in abstract	Three injections of 6 mL, 2 weeks apart	Plaque size decreased from 11.1 mm to 8.2 mm (*p* = 0.004); curvature decreased from 50° to 40° (*p* < 0.001); PDQ domains improved; IIEF-5 not significantly changed (*p* = 0.3)	No adverse events/side effects reported	Prospective design; relatively large PD cohort; objective plaque and curvature outcomes; PDQ included	No comparator; short-term follow-up; PRP composition incomplete; erectile function not improved
Ergün and Sağır, 2025 [60]	Chronic-phase PD, disease duration ≥ 1 year	Retrospective single-arm combination study; *n* = 26	20 mL blood; double-spin (980 g then 1900 g); 2 mL PRP obtained	3 PRP + 6 Li-ESWT sessions over 3 weeks; no control arm	Mean curvature improved from 40.25° ± 10.57° to 30.15° ± 11.91° (*p* < 0.05); plaque size change not significant; 53.8% satisfied	Mild bruising in 2; 1 new plaque elsewhere; no major adverse events	Clearly defined chronic-phase cohort; protocol details provided	Combination therapy prevents attribution to PRP alone; retrospective; small sample; no validated PD questionnaires
Karakose and Yitgin, 2024 [61]	Acute-phase PD	Retrospective comparative study; *n* = 159 (group 1 oral therapy *n* = 77; group 2 Li-ESWT + PRP + tadalafil *n* = 82)	30 cc blood; two centrifugations at 1200 RCF; 3 cc PRP	Group 2 received Li-ESWT + PRP weekly for 6 weeks + daily tadalafil; group 1 received vitamin E + colchicine + tadalafil	Greater improvement in group 2 vs. group 1 in plaque size (−6.1 vs. −1.1 mm), curvature (−10.5° vs. −4.5°), IIEF-5, and VAS; all *p* < 0.001 between groups	No ecchymosis/hematoma reported in intervention group; no systemic reactions	Comparative design; larger acute-phase cohort; includes pain and erectile outcomes	Retrospective; multimodal intervention; non-randomized; comparator not placebo; impossible to isolate PRP contribution
Ledesma et al., 2024 [62]	PD patients; phase not explicitly stated	Phase 2 randomized, placebo-controlled crossover trial; *n* = 41 randomized, preliminary analysis reported for 28 patients	PRFM via Arthrex Angel + calcium chloride	Intralesional penile injections; 2 injections of PRP or placebo over 3 months, followed by crossover to 2 injections of alternate treatment over the next 3 months	Preliminary results suggested no significant change in IIEF scores; PDQ score decreased significantly only in the placebo–PRP sequence, not in the PRP–placebo sequence; baseline median curvature was 40° in both groups; at 6 months, curvature reduction was significant in the PRP–placebo group (40° to 25°, *p* = 0.047) but not in the placebo–PRP group (40° to 32.5°, *p* = 0.490); authors interpreted findings as suggesting a possible delayed PRP effect	No adverse events, including no penile complications, reported during follow-up	Randomized placebo-controlled design; crossover structure allowed within-study comparison; included pain, curvature, PDQ, and erectile function outcomes; favorable short-term safety profile	Preliminary analysis only; small effective sample size; crossover design complicates interpretation of durability and carryover effects; incomplete PRP biologic characterization; efficacy signal inconsistent across outcomes

Abbreviations: Li-ESWT, low-intensity extracorporeal shockwave therapy; PD, Peyronie’s disease; PDQ, Peyronie’s Disease Questionnaire; PRFM, platelet-rich fibrin matrix; PRP, platelet-rich plasma.

**Table 4:** Structured evidence table of PRP for interstitial cystitis/bladder pain syndrome.

Study	Population	Design/Sample Size	PRP Preparation/Characterization	Delivery Protocol/Comparator	Main Efficacy Findings	Safety Findings	Strengths	Limitations
Mourad et al., 2025 [64]	IC/PBS refractory to conventional treatment	Prospective one-arm clinical trial; *n* = 30 enrolled, *n* = 26 analyzed	50 mL blood; double-spin (100 g × 15 min, then 1600 g × 20 min); 10 mL PRP, estimated ~1,000,000 platelets/µL (~4× baseline)	Single session, 20 submucosal injections of 0.5 mL; posterior and lateral bladder walls	At 6 months, 16/26 (61.5%) achieved success (GRA ≥ 2); significant reductions in ICSI, ICPI, pain VAS, frequency, and nocturia; FBC increased from 139.7 ± 6.3 to 290.1 ± 71.3 mL	Hematuria in 11.5%, UTI in 15.4%	Clearly described PRP preparation and injection technique; includes prespecified success definition and NRI sensitivity	No control group; complete-case analysis primary; modest sample; single-session design may limit durability
Zheng et al., 2025 [65]	Non-Hunner IC/BPS refractory to ≥3 treatments	Prospective protocol/feasibility study; *n* = 17	Blood cell separator protocol; mean platelet enrichment coefficient 5.11 ± 1.27; mean PRP collection volume 125.1 ± 17.5 mL; aliquoted and cryopreserved	Intended 6 intravesical submucosal injections; one bag same day, remainder stored at −80 °C	Symptom improvement in 12/17 (70.6%) by GRA ≥ 5; O’Leary–Sant and VAS improved significantly; no change in several diary metrics	No collection-related adverse events; one gross hematuria after 4th injection	Valuable protocol standardization paper; detailed collection logistics; biologic enrichment reported	Small exploratory cohort; variable completion of injection course; no comparator; focus partly on collection feasibility rather than efficacy
Jhang et al., 2023 [63]	IC/BPS refractory to conventional treatment	Prospective clinical trial	Prior standard PRP method	Repeated intravesical PRP injections	Reported symptom improvement and safety	Generally acceptable short-term safety	Foundational human IC/BPS PRP study	Earlier small uncontrolled design

Abbreviations: FBC, functional bladder capacity; GRA, Global Response Assessment; IC/BPS, interstitial cystitis/bladder pain syndrome; ICPI, Interstitial Cystitis Problem Index; ICSI, Interstitial Cystitis Symptom Index; PRP, platelet-rich plasma; rUTI, recurrent urinary tract infection; VAS, visual analog scale.

**Table 5:** Structured evidence table of PRP for stress urinary incontinence.

Study	Population	Design/Sample Size	PRP Preparation/Characterization	Delivery Protocol/Comparator	Main Efficacy Findings	Safety Findings	Strengths	Limitations
Long et al., 2021 [69]	Women with mild-to-severe SUI	Prospective pilot interventional study; *n* = 20	RegenKit; 20 mL whole blood; ~10 mL total PRP produced; platelet concentration ~1.6× baseline	Anterior vaginal wall/mid-urethral region; 5 mL/session; monthly ×3	Significant improvement in ICIQ-SF, UDI-6, and IIQ-7 at 1 and 6 months; overall efficacy 12/20 (60%) improved/cured	No adverse reactions reported	First dedicated female SUI PRP pilot; complete 6-month follow-up	No control group; small sample; mainly questionnaire-based outcomes; low platelet enrichment
Jiang et al., 2021 [70]	Men and women with SUI due to urodynamically proven intrinsic sphincter deficiency	Prospective proof-of-concept trial; *n* = 35	50 mL blood; double-spin; PRP platelet concentration 2.5–5× whole blood	Urethral sphincter injection, 5 mL at 5 sites, monthly ×4	Complete dryness in 20%; moderate improvement in 40%; total success 60%; VAS improved from 6.57 ± 1.89 to 3.77 ± 2.41; ALPP increased from 98.3 ± 55.8 to 157.3 ± 79.3 cmH_2_O	Mild hematuria/dysuria in 28.6%, resolved conservatively; no UTI or retention	Includes objective urodynamics; mechanistically targeted ISD population	No control group; mixed etiologies; lower efficacy in neurogenic SUI
Athanasiou et al., 2021 [71]	Female SUI	Prospective pilot	RegenKit	Anterior vaginal wall/periurethral, 5.5 mL, twice in 4–6 weeks	ICIQ-FLUTS and 1 h pad test improved	No major safety signal emphasized	Includes objective pad test	Small uncontrolled study
Grigoriadis et al., 2024 [72]	Women with SUI and urodynamic stress incontinence	Double-blind randomized sham-controlled trial; *n* = 50	Autologous PRP; biologic characterization incompletely reported in abstract	Periurethral injection at 3 urethral levels; 2 sessions, 4- to 6-week intervals; sham saline comparator	Significant improvement in subjective symptoms versus sham; subjective cure 32% vs. 4%; significant reduction in 1 h pad test urine loss at 6 months	No adverse events reported	High-quality sham-controlled design; included objective pad test and validated symptom questionnaires	Small single-center trial; limited PRP processing detail; no long-term follow-up beyond 6 months
Ashton et al., 2024 [73]	Women with stress-predominant urinary incontinence	Single-blind randomized placebo-controlled trial; *n* = 50	5 mL autologous PRP; biologic characterization incompletely reported	Single mid-urethral anterior vaginal wall injection vs. saline placebo	No significant difference in composite treatment success at 6 months	Minor adverse events, similar between groups	Placebo-controlled randomized design with objective and subjective endpoint components	Small single-center study; single-blind design; limited PRP reporting; one-time injection protocol may have been insufficient
Liu et al., 2022 [68]	Rat postpartum SUI model	Preclinical animal study; control *n* = 10, PSUI *n* = 10, PSUI+PRP *n* = 10	Autologous PRP to pelvic floor muscles	Ultrasound-guided pelvic floor PRP injection	BLPP and ALPP significantly improved; PRP attenuated proprioceptor abnormalities and increased NT-3/MY-32 expression	Preclinical safety only	Supports mechanistic plausibility	Not human evidence

Abbreviations: ALPP, abdominal leak point pressure; BLPP, bladder leak point pressure; ICIQ-SF, International Consultation on Incontinence Questionnaire–Short Form; ISD, intrinsic sphincter deficiency; PRP, platelet-rich plasma; SUI, stress urinary incontinence; UDI-6, Urogenital Distress Inventory-6.

**Table 6:** Minimum PRP-Uro Checklist for clinical studies of platelet-rich plasma in urology and sexual medicine.

Domain	Reporting Item	Purpose
Study design	Study design and comparator	Defines internal validity and clinical interpretability
	Trial registration and/or protocol availability	Reduces selective reporting
	Prespecified primary and secondary endpoints	Clarifies hypothesis-driven outcome assessment
	Sample size justification	Helps assess statistical robustness
Population	Diagnostic criteria	Ensures cross-study comparability
	Disease subtype or phase	Captures biologically relevant heterogeneity
	Baseline severity and symptom duration	Supports case-mix interpretation
	Prior and concomitant therapies	Identifies major confounders
PRP source and processing	Whole-blood collection volume	Enables estimation of biologic dose
	Device/kit and manufacturer	Major determinant of product variability
	Centrifugation parameters	Essential for reproducibility
	Anticoagulant and activation method	Influences PRP composition and growth factor release
	Final PRP volume	Required for dose standardization
	Platelet count in whole blood and PRP	Minimum biologic characterization
	Platelet enrichment ratio and, ideally, absolute platelet dose	Improves dose comparability
	Leukocyte and erythrocyte content	Distinguishes PRP subtype
	Storage conditions	Important for repeated-treatment protocols
Delivery protocol	Anatomical target and route	Determines mechanistic plausibility and safety
	Number of injections, interval, and total course	Major source of protocol heterogeneity
	Volume per site and per session	Required for reproducibility
	Guidance and anesthesia	Affects accuracy and tolerability
	Co-interventions	Essential for attribution of efficacy
Outcome assessment	Validated disease-specific instruments	Improves interpretability
	Objective endpoints where applicable	Reduces reliance on subjective outcomes alone
	Follow-up timepoints and durability	Captures persistence of effect
	Definition of treatment success/MCID/responder threshold	Prevents post hoc outcome inflation
Safety	Immediate procedural complications	Core procedural safety reporting
	Delayed adverse events and severity	Supports balanced risk–benefit assessment
	Indication-specific adverse events	Improves clinical relevance
Analysis and transparency	Missing data and attrition handling	Important in small regenerative medicine studies
	Between-group comparisons where applicable	Strengthens causal inference
	Conflicts of interest and device involvement	Important in procedure- and device-dependent interventions

The PRP-Uro Checklist is proposed as a practical minimum reporting framework for platelet-rich plasma studies in urology and sexual medicine. It is intended to improve reproducibility, facilitate cross-study comparison, and support future evidence synthesis. In contrast to generic PRP reporting recommendations, PRP-Uro incorporates indication-specific elements that are particularly relevant to urologic and sexual medicine applications, including disease phase or subtype, injection route and anatomical target, procedure-related guidance, co-interventions, and objective functional endpoints. Examples of indication-specific objective outcomes include duplex ultrasound in erectile dysfunction, standardized curvature assessment in Peyronie’s disease, pad testing and abdominal leak point pressure in stress urinary incontinence, and bladder diary variables, functional bladder capacity, cystoscopic findings, or urothelial biomarkers in interstitial cystitis/bladder pain syndrome. Abbreviations: MCID, minimal clinically important difference; PRP, platelet-rich plasma.

## Data Availability

No new data were created or analyzed in this study. Data sharing is not applicable to this article.

## Abbreviations

The following abbreviations are used in this manuscript:

PRP	Platelet-Rich Plasma
ED	Erectile Dysfunction
PD	Peyronie’s Disease
SUI	Stress Urinary Incontinence
IC/BPS	Interstitial Cystitis/Bladder Pain Syndrome
PDE5i	Phosphodiesterase Type 5 Inhibitors
Li-ESWT	Low-Intensity Extracorporeal Shockwave Therapy
IIEF	International Index of Erectile Function
IIEF-EF	International Index of Erectile Function—Erectile Function domain
EHS	Erection Hardness Score
PSV	Peak Systolic Velocity
EDV	End-Diastolic Velocity
ICP/MAP	Intracavernosal Pressure/Mean Arterial Pressure
VEGF	Vascular Endothelial Growth Factor
TGF-β	Transforming Growth Factor-beta
PDGF	Platelet-Derived Growth Factor
IGF-1	Insulin-like Growth Factor-1
BDNF	Brain-Derived Neurotrophic Factor
ECM	Extracellular Matrix
MSCs	Mesenchymal Stem Cells
EVs	Extracellular Vesicles
TNF-α	Tumor Necrosis Factor-alpha
GSM	Genitourinary Syndrome of Menopause
POP	Pelvic Organ Prolapse
SSCs	Spermatogonial Stem Cells
RCT	Randomized Controlled Trial
MCID	Minimal Clinically Important Difference
P-PRP	Pure Platelet-Rich Plasma
L-PRP	Leukocyte- and Platelet-Rich Plasma
P-PRF	Pure Platelet-Rich Fibrin
L-PRF	Leukocyte- and Platelet-Rich Fibrin
RCF	Relative Centrifugal Force
Wnt	Wingless-type MMTV integration site family
proNGF	Pro-Nerve Growth Factor
CXCL5	C-X-C Motif Chemokine Ligand 5
S1PR1	Sphingosine-1-Phosphate Receptor 1
PI3K/Akt	Phosphoinositide 3-Kinase/Protein Kinase B

## Appendix A. Search Strategies

((platelet-rich plasma) OR (PRP)) AND ((erectile dysfunction) OR (Peyronie*) OR (stress urinary incontinence) OR (interstitial cystitis) OR (bladder pain syndrome) OR (urology) OR (andrology) OR (sexual medicine))

(“platelet rich plasma”[MeSH Terms] OR (“platelet rich”[All Fields] AND “plasma”[All Fields]) OR “platelet rich plasma”[All Fields] OR (“platelet”[All Fields] AND “rich”[All Fields] AND “plasma”[All Fields]) OR “platelet rich plasma”[All Fields] OR (“pharmacol res perspect”[Journal] OR “prp”[All Fields])) AND (“erectile dysfunction”[MeSH Terms] OR (“erectile”[All Fields] AND “dysfunction”[All Fields]) OR “erectile dysfunction”[All Fields] OR “peyronie*”[All Fields] OR (“urinary incontinence, stress”[MeSH Terms] OR (“urinary”[All Fields] AND “incontinence”[All Fields] AND “stress”[All Fields]) OR “stress urinary incontinence”[All Fields] OR (“stress”[All Fields] AND “urinary”[All Fields] AND “incontinence”[All Fields])) OR (“cystitis, interstitial”[MeSH Terms] OR (“cystitis”[All Fields] AND “interstitial”[All Fields]) OR “interstitial cystitis”[All Fields] OR (“interstitial”[All Fields] AND “cystitis”[All Fields])) OR (“cystitis, interstitial”[MeSH Terms] OR (“cystitis”[All Fields] AND “interstitial”[All Fields]) OR “interstitial cystitis”[All Fields] OR (“bladder”[All Fields] AND “pain”[All Fields] AND “syndrome”[All Fields]) OR “bladder pain syndrome”[All Fields]) OR (“urologie”[All Fields] OR “urology”[MeSH Terms] OR “urology”[All Fields] OR “urology s”[All Fields]) OR (“andrology”[MeSH Terms] OR “andrology”[All Fields]) OR (“sexology”[MeSH Terms] OR “sexology”[All Fields] OR (“sexual”[All Fields] AND “medicine”[All Fields]) OR “sexual medicine”[All Fields])).

## Appendix B. Search Filter Details

Filters applied: Adaptive Clinical Trial, Clinical Study, Clinical Trial, Clinical Trial, Phase I, Clinical Trial, Phase II, Clinical Trial, Phase III, Clinical Trial, Phase IV, Comparative Study, Controlled Clinical Trial, English Abstract, Meta-Analysis, Multicenter Study, Network Meta-Analysis, Observational Study, Randomized Controlled Trial, Review, Scoping Review, Systematic Review, from 1 January 2021 to 30 October 2025.

## References

[1] Anastasiadis E., Ahmed R., Khoja A.K., Yap T. (2022). Erectile dysfunction: Is platelet-rich plasma the new frontier for treatment in patients with erectile dysfunction? A review of the existing evidence. Front. Reprod. Health.

[2] Castiglione F., Cakir O.O., Satchi M., Fallara G., Pang K.H., European Society for Sexual Medicine Scientific Collaboration and Partnership (2023). The Current Role and Implications of Stem Cell Therapy in Erectile Dysfunction: A Transformation from Caterpillar to Butterfly Is Required. Eur. Urol. Focus.

[3] Cecerska-Heryć E., Goszka M., Serwin N., Roszak M., Grygorcewicz B., Heryć R., Dołęgowska B. (2022). Applications of the regenerative capacity of platelets in modern medicine. Cytokine Growth Factor Rev..

[4] Huang Y.C., Wu C.T., Chen M.F., Kuo Y.H., Li J.M., Shi C.S. (2021). Intracavernous injection of autologous platelet-rich plasma ameliorates hyperlipidemia-associated erectile dysfunction in a rat model. Sex. Med..

[5] Lim J.J., Belk J.W., Wharton B.R., McCarthy T.P., McCarty E.C., Dragoo J.L., Frank R.M. (2025). Most orthopaedic platelet-rich plasma investigations don’t report protocols and composition: An updated systematic review. Arthroscopy.

[6] Rahman E., Rao P., Abu-Farsakh H.N., Thonse C., Ali I., Upton A.E., Baratikkae S.Y., Carruthers J.D.A., Mosahebi A., Heidari N. (2024). Systematic review of platelet-rich plasma in medical and surgical specialties: Quality, evaluation, evidence, and enforcement. J. Clin. Med..

[7] Marín Fermín T., Calcei J.G., Della Vedova F., Martinez Cano J.P., Arias Calderon C., Imam M.A., Khoury M., Laupheimer M.W., D’Hooghe P. (2024). Review of Dohan Ehrenfest et al. (2009) on “Classification of platelet concentrates: From pure platelet-rich plasma (P-PRP) to leucocyte- and platelet-rich fibrin (L-PRF)”. J. ISAKOS.

[8] Saqlain N., Mazher N., Fateen T., Siddique A. (2023). Comparison of single and double centrifugation methods for preparation of platelet-rich plasma (PRP). Pak. J. Med. Sci..

[9] Turajane T., Cheeva-Akrapan V., Saengsirinavin P., Lappaiwong W. (2023). Composition of platelet-rich plasma prepared from knee osteoarthritic patients: Platelets, leukocytes, and subtypes of leukocyte. Cureus.

[10] Gentile P., Calabrese C., De Angelis B., Dionisi L., Pizzicannella J., Kothari A., De Fazio D., Garcovich S. (2020). Impact of the different preparation methods to obtain autologous non-activated platelet-rich plasma (A-PRP) and activated platelet-rich plasma (AA-PRP) in plastic surgery: Wound healing and hair regrowth evaluation. Int. J. Mol. Sci..

[11] Melnikov D.V., Kirillova K.A., Zakharenko A.S., Sinelnikov M.Y., Ragimov A.A., Istranov A.L., Startseva O.I. (2021). Effect of cryo-processing on platelet-rich autoplasma preparations. Sovrem. Tehnol. Med..

[12] Mercader Ruiz J., Beitia M., Delgado D., Sánchez P., Sánchez M.B., Oraa J., Benito-Lopez F., Basabe-Desmonts L., Sánchez M. (2024). Method to obtain a plasma rich in platelet- and plasma-growth factors based on water evaporation. PLoS ONE.

[13] Wu W.S., Chen L.R., Chen K.H. (2025). Platelet-rich plasma (PRP): Molecular mechanisms, actions and clinical applications in human body. Int. J. Mol. Sci..

[14] Del Amo C., Perez-Valle A., Atilano L., Andia I. (2022). Unraveling the signaling secretome of platelet-rich plasma: Towards a better understanding of its therapeutic potential in knee osteoarthritis. J. Clin. Med..

[15] Fidan B.B., Koç E., Özotuk E.Ç., Kaplan O., Çelebier M., Korkusuz F. (2025). Do preparation techniques transform the metabolite profile of platelet-rich plasma?. Bioengineering.

[16] Le N.T.N., Han C.L., Delila L., Nebie O., Chien H.T., Wu Y.W., Buée L., Blum D., Burnouf T. (2024). Proteomics of human platelet lysates and insight from animal studies on platelet protein diffusion to hippocampus upon intranasal administration. APL Bioeng..

[17] Wang S.L., Liu X.L., Kang Z.C., Wang Y.S. (2023). Platelet-rich plasma promotes peripheral nerve regeneration after sciatic nerve injury. Neural Regen. Res..

[18] Kim J.W., Kim J.M., Choi M.E., Jeon E.J., Park J.M., Kim Y.M., Choi S.H., Eom T., Shim B.S., Choi J.S. (2022). Platelet-rich plasma loaded nerve guidance conduit as implantable biocompatible materials for recurrent laryngeal nerve regeneration. NPJ Regen. Med..

[19] Winias S., Sarasati A., Wicaksono S., Ayuningtyas N.F., Ernawati D.S., Radithia D. (2024). BDNF and Krox20 as indicators of platelet-rich plasma-induced nerve regeneration in a neuropathic orofacial pain model. Eur. J. Dent..

[20] Zhang Y., Yi D., Hong Q., Cao J., Geng X., Liu J., Xu C., Cao M., Chen C., Xu S. (2024). Platelet-rich plasma-derived exosomes boost mesenchymal stem cells to promote peripheral nerve regeneration. J. Control. Release.

[21] Zhang Y., Yi D., Hong Q., Liu C., Chi K., Liu J., Li X., Ye Y., Zhu Y., Peng N. (2024). Platelet-rich plasma-derived exosomes enhance mesenchymal stem cell paracrine function and nerve regeneration potential. Biochem. Biophys. Res. Commun..

[22] Ismy J., Khalilullah S.A., Maulana R., Hidayatullah F. (2024). A potential treatment for erectile dysfunction: Effect of platelet-rich plasma administration on axon and collagen regeneration in cavernous nerve injury. Narra J.

[23] Kamayana J.A.S., Hamid A.R.R.H., Mahadewa T.G.B., Sanjaya I.G.P.H., Darmajaya I.M., Dewi I.G.A.S.M. (2024). Preconditioning local injection of activated platelet-rich plasma increases angiogenesis, VEGF levels, and viability of modified McFarlane flap in diabetes-induced rats. Arch. Plast. Surg..

[24] Chen T., Song P., He M., Rui S., Duan X., Ma Y., Armstrong D.G., Deng W. (2023). Sphingosine-1-phosphate derived from PRP-Exos promotes angiogenesis in diabetic wound healing via the S1PR1/AKT/FN1 signalling pathway. Burn. Trauma.

[25] Masterson T.A., Molina M., Ledesma B., Zucker I., Saltzman R., Ibrahim E., Han S., Reis I.M., Ramasamy R. (2023). Platelet-rich plasma for the treatment of erectile dysfunction: A prospective, randomized, double-blind, placebo-controlled clinical trial. J. Urol..

[26] Ragheb A.M., Lotfy A.M., Fahmy M., Elmarakbi A.A. (2024). Safety and efficacy of platelet-rich plasma injection for treatment of erectile dysfunction: A prospective randomized controlled study. Basic Clin. Androl..

[27] Asmundo M.G., Durukan E., von Rohden E., Thy S.A., Jensen C.F.S., Fode M. (2024). Platelet-rich plasma therapy in erectile dysfunction and Peyronie’s disease: A systematic review of the literature. World J. Urol..

[28] Berndt S., Carpentier G., Turzi A., Borlat F., Cuendet M., Modarressi A. (2021). Angiogenesis is differentially modulated by platelet-derived products. Biomedicines.

[29] Wang H.C., Li Z., Li Z., Wang X., Long X. (2023). Platelet-rich plasma combined fat transplantation for the treatment of bleomycin-induced murine scleroderma. Ann. Plast. Surg..

[30] Stilhano R.S., Denapoli P.M.A., Gallo C.C., Samoto V.Y., Ingham S.J.M., Abdalla R.J., Koh T.J., Han S.W. (2021). Regenerative effect of platelet-rich plasma in the murine ischemic limbs. Life Sci..

[31] Yadav A., Ramasamy T.S., Lin S.C., Chen S.H., Lu J., Liu Y.H., Lu F.I., Hsueh Y.Y., Lin S.P., Wu C.C. (2022). Autologous platelet-rich growth factor reduces M1 macrophages and modulates inflammatory microenvironments to promote sciatic nerve regeneration. Biomedicines.

[32] Dong Q., Yang X., Liang X., Liu J., Wang B., Zhao Y., Huselstein C., Feng X., Tong Z., Chen Y. (2023). Composite hydrogel conduit incorporated with platelet-rich plasma improved the regenerative microenvironment for peripheral nerve repair. ACS Appl. Mater. Interfaces.

[33] Dai P., Wu Y., Gao Y., Li M., Zhu M., Xu H., Feng X., Jin Y., Zhang X. (2024). Multiomics analysis of platelet-rich plasma promoting biological performance of mesenchymal stem cells. BMC Genom..

[34] Chen E., Chen Z., Chen L., Hu X. (2022). Platelet-derived respiratory-competent mitochondria transfer to mesenchymal stem cells to promote wound healing via metabolic reprogramming. Platelets.

[35] Khodamoradi K., Dullea A., Golan R., Molina M., Arora H., Masterson T.A., Ramasamy R. (2022). Platelet rich plasma (PRP) growth factor concentration varies in men with erectile dysfunction. J. Sex. Med..

[36] Shaher H., Fathi A., Elbashir S., Abdelbaki S.A., Soliman T. (2023). Is platelet rich plasma safe and effective in treatment of erectile dysfunction? Randomized controlled study. Urology.

[37] Huang H., Qin J., Wen Z., Liu Y., Chen C., Wang C., Li H., Yang X. (2024). Efficacy and safety of platelet-rich plasma (PRP) in erectile dysfunction (ED): A systematic review and meta-analysis. Transl. Androl. Urol..

[38] Poulios E., Mykoniatis I., Pyrgidis N., Zilotis F., Kapoteli P., Kotsiris D., Kalyvianakis D., Hatzichristou D. (2021). Platelet-rich plasma (PRP) improves erectile function: A double-blind, randomized, placebo-controlled clinical trial. J. Sex. Med..

[39] Francomano D., Iuliano S., Dehò F., Capogrosso P., Tuzzolo P., LAVignera S., Antonini G., Aversa A. (2025). Regenerative treatment with platelet-rich plasma in patients with refractory erectile dysfunction: Short-term outcomes and predictive value of mean platelet volume. Minerva Endocrinol..

[40] Suharyani S., Leonardo M., Oentoeng H.H., Pardamean Lumban Tobing E.R., Tansol C., Hariyanto T.I. (2024). Efficacy and safety of platelet-rich plasma intracavernous injection for patients with erectile dysfunction: A systematic review, meta-analysis, and meta-regression. Asian J. Urol..

[41] Taş T., Çakıroğlu B., Arda E., Onuk Ö., Nuhoğlu B. (2021). Early clinical results of the tolerability, safety, and efficacy of autologous platelet-rich plasma administration in erectile dysfunction. Sex. Med..

[42] Zaghloul A.S., El-Nashaar A.M., Said S.Z., Osman I.A., Mostafa T. (2022). Assessment of the intracavernosal injection platelet-rich plasma in addition to daily oral tadalafil intake in diabetic patients with erectile dysfunction non-responding to on-demand oral PDE5 inhibitors. Andrologia.

[43] Zaghloul A.S., Mahmoud ElNashar A.E.R., GamalEl Din S.F., Zaki Said S., Saad H.M., Refaat Eldebs H., Abdel Latif Osman I. (2021). Smoking status and the baseline international index of erectile function score can predict satisfactory response to platelet-rich plasma in patients with erectile dysfunction: A prospective pilot study. Andrologia.

[44] Zhou Z., Wang Y., Chai Y., Wang T., Yan P., Zhang Y., Yang X. (2025). The efficacy of platelet-rich plasma (PRP) alone or in combination with low intensity shock wave therapy (Li-SWT) in treating erectile dysfunction: A systematic review and meta-analysis of seven randomized controlled trials. Aging Male.

[45] Dachille G., Panunzio A., Bizzotto L., D’Agostino M.V., Greco F., Guglielmi G., Carbonara U., Spilotros M., Citarella C., Ostuni A. (2025). Platelet-rich plasma intra-plaque injections rapidly reduce penile curvature and improve sexual function in Peyronie’s disease patients: Results from a prospective large-cohort study. World J. Urol..

[46] Matz E.L., Scarberry K., Terlecki R. (2022). Platelet-rich plasma and cellular therapies for sexual medicine and beyond. Sex. Med. Rev..

[47] Poulios E., Mykoniatis I., Pyrgidis N., Kalyvianakis D., Hatzichristou D. (2023). Platelet-rich plasma for the treatment of erectile dysfunction: A systematic review of preclinical and clinical studies. Sex. Med. Rev..

[48] Akakpo W., Schirmann A., Ferretti L., Ben-Naoum K., Carnicelli D., Graziana J.P., Hupertan V., Madec F.X., Marcelli F., Methorst C. (2020). Biotherapies for erectile dysfunction and Peyronie’s disease: Where are we now?. Prog. Urol..

[49] Yang W., Qiu C., Zhai J., Zhang W., Huang C., Shao J., Zhang J., Chen S., Miao X., Chen P. (2023). Ultrasound-targeted microbubble destruction mediates PDE5i/NO integration for cavernosum remodeling and penile rehabilitation. Bioeng. Transl. Med..

[50] Chung D.Y., Song K.M., Choi M.J., Limanjaya A., Ghatak K., Ock J., Yin G.N., Hong C.H., Hong S.S., Suh J.K. (2021). Neutralizing antibody to proNGF rescues erectile function by regulating the expression of neurotrophic and angiogenic factors in a mouse model of cavernous nerve injury. Andrology.

[51] Gu X., Thakker P.U., Matz E.L., Terlecki R.P., Marini F.C., Allickson J.G., Lue T.F., Lin G., Atala A., Yoo J.J. (2020). Dynamic changes in erectile function and histological architecture after intracorporal injection of human placental stem cells in a pelvic neurovascular injury rat model. J. Sex. Med..

[52] Wang W., Liu Y., Zhou Z.H., Pang K., Wang J.K., Huan P.F., Lu J.R., Zhu T., Zhu Z.B., Han C.H. (2025). Effects of human umbilical cord-derived mesenchymal stem cell therapy for cavernous nerve injury-induced erectile dysfunction in the rat model. Asian J. Androl..

[53] Shao J., Nie P., Yang W., Guo R., Ding D., Liang R., Wei B., Wei H. (2022). An EPO-loaded multifunctional hydrogel synergizing with adipose-derived stem cells restores neurogenic erectile function via enhancing nerve regeneration and penile rehabilitation. Bioeng. Transl. Med..

[54] Kim J.H., Yun J.H., Song E.S., Kim S.U., Lee H.J., Song Y.S. (2021). Improvement of damaged cavernosa followed by neuron-like differentiation at injured cavernous nerve after transplantation of stem cells seeded on the PLA nanofiber in rats with cavernous nerve injury. Mol. Biol. Rep..

[55] Gu S.J., Li M., Yuan Y.M., Xin Z.C., Guan R.L. (2021). A novel flavonoid derivative of icariside II improves erectile dysfunction in a rat model of cavernous nerve injury. Andrology.

[56] Wu Y.N., Liao C.H., Chen K.C., Chiang H.S. (2021). CXCL5 cytokine is a major factor in platelet-rich plasma’s preservation of erectile function in rats after bilateral cavernous nerve injury. J. Sex. Med..

[57] Liao C.H., Lee K.H., Chung S.D., Chen K.C., Praveen Rajneesh C., Chen B.H., Cheng J.H., Lin W.Y., Chiang H.S., Wu Y.N. (2022). Intracavernous injection of platelet-rich plasma therapy enhances erectile function and decreases the mortality rate in streptozotocin-induced diabetic rats. Int. J. Mol. Sci..

[58] Matthew A.N., Rogers D.E., Grob G., Blottner M., Kodama S., Krzastek S.C. (2023). The use of low-intensity extracorporeal shockwave therapy in management of erectile dysfunction following prostate cancer treatment: A review of the current literature. Transl. Androl. Urol..

[59] Dogan K., Cil G. (2025). Erectile function recovery using shockwave and platelet-rich plasma: A single-centre prospective comparative study. Arch. Esp. Urol..

[60] Ergün M., Sağır S. (2025). Low-intensity extracorporeal shock wave therapy and platelet-rich plasma: Effective combination treatment of chronic-phase Peyronie’s disease. Arch. Esp. Urol..

[61] Karakose A., Yitgin Y. (2024). A new alternative approach to management of acute phase Peyronie’s disease: Low intensity extracorporeal shockwave therapy and platelet-rich plasma. Minerva Urol. Nephrol..

[62] Ledesma B.R., Velasquez D.A., Egemba C., Molina M., Ibrahim E., Costantini-Mesquita F., Deebel N.A., Han S., Reis I.M., Saltzman R. (2024). A phase 2 randomized, placebo-controlled crossover trial to evaluate safety and efficacy of platelet-rich plasma injections for Peyronie’s disease: Clinical trial update. Int. J. Impot. Res..

[63] Jhang J.F., Jiang Y.H., Lin T.Y., Kuo H.C. (2023). The tumor necrosis factor-α level in platelet-rich plasma might be associated with treatment outcome in patients with interstitial cystitis/bladder pain syndrome or recurrent urinary tract infection. Int. J. Mol. Sci..

[64] Mourad M.S., Tawfick A., Kotb M., Saleh I.M., Salim M.S., Samir Y.R. (2026). Efficacy and safety of submucosal intravesical injection of platelet-rich plasma in the treatment of interstitial cystitis/painful bladder syndrome. Int. Urogynecol. J..

[65] Zheng Q., Zhang P., Guo W., Zhang J., Zhang F., Ma C., Yang Y., Cui L., Wu Y., Zhang L. (2025). A standardized protocol for modified platelet-rich plasma collection for the treatment of interstitial cystitis/bladder pain syndrome. Bladder.

[66] Chen A.H., Trabuco E.C., Chumsri S., Thielen J.M., Cornella J.L., Shapiro S.A., Heckman M.G., Dukes R.E., Arthurs J.R., Blumenfeld S.G. (2025). Platelet-rich plasma for genitourinary syndrome of menopause in breast cancer survivors. Obstet. Gynecol..

[67] Sacarin G., Abu-Awwad A., Razvan N., Craina M., Hogea B., Sorop B., Abu-Awwad S.A., Diaconu M., Pilut N.C., Suba M.I. (2025). Sexual quality of life in postmenopausal women: A comparative randomized controlled trial of intravaginal PRP therapy versus local hormonal treatments. Medicina.

[68] Liu J., Liu Z., Tang Y., Munoz A., Zhang Y., Li X. (2022). Treatment with platelet-rich plasma attenuates proprioceptor abnormalities in a rat model of postpartum stress urinary incontinence. Int. Urogynecol. J..

[69] Long C.Y., Lin K.L., Shen C.R., Ker C.R., Liu Y.Y., Loo Z.X., Hsiao H.H., Lee Y.C. (2021). A pilot study: Effectiveness of local injection of autologous platelet-rich plasma in treating women with stress urinary incontinence. Sci. Rep..

[70] Jiang Y.H., Lee P.J., Kuo H.C. (2021). Therapeutic efficacy of urethral sphincter injections of platelet-rich plasma for the treatment of stress urinary incontinence due to intrinsic sphincter deficiency: A proof-of-concept clinical trial. Int. Neurourol. J..

[71] Athanasiou S., Kalantzis C., Zacharakis D., Kathopoulis N., Pontikaki A., Grigoriadis T. (2021). The Use of Platelet-rich Plasma as a Novel Nonsurgical Treatment of the Female Stress Urinary Incontinence: A Prospective Pilot Study. Female Pelvic Med. Reconstr. Surg..

[72] Grigoriadis T., Kalantzis C., Zacharakis D., Kathopoulis N., Prodromidou A., Xadzilia S., Athanasiou S. (2024). Platelet-Rich Plasma for the Treatment of Stress Urinary Incontinence—A Randomized Trial. Urogynecology.

[73] Ashton L., Nakatsuka H., Johnson C.M., Kenne K., Kreder K.J., Kruse R., Wendt L., Takacs E.B., Vollstedt A.J. (2024). A Single Injection of Platelet-rich Plasma Injection for the Treatment of Stress Urinary Incontinence in Females: A Randomized Placebo-controlled Trial. Urology.

[74] Prodromidou A., Zacharakis D., Athanasiou S., Protopapas A., Michala L., Kathopoulis N., Grigoriadis T. (2022). The emerging role on the use of platelet-rich plasma products in the management of urogynaecological disorders. Surg. Innov..

[75] De Ponte A., Cabrera S., Sparice S.S.B., Baulies S., Rodríguez I. (2026). Platelet-rich plasma in the management of vulvovaginal disorders: A systematic review. J. Sex. Med..

[76] Villalpando B.K., Wyles S.P., Schaefer L.A., Bodiford K.J., Bruce A.J. (2021). Platelet-rich plasma for the treatment of lichen sclerosus. Plast. Aesthetic Res..

[77] Paganelli A., Contu L., Condorelli A., Ficarelli E., Motolese A., Paganelli R., Motolese A. (2023). Platelet-rich plasma (PRP) and adipose-derived stem cell (ADSC) therapy in the treatment of genital lichen sclerosus: A comprehensive review. Int. J. Mol. Sci..

[78] Dankova I., Pyrgidis N., Tishukov M., Georgiadou E., Nigdelis M.P., Solomayer E.F., Marcon J., Stief C.G., Hatzichristou D. (2023). Efficacy and safety of platelet-rich plasma injections for the treatment of female sexual dysfunction and stress urinary incontinence: A systematic review. Biomedicines.

[79] Khadivi F., Koruji M., Akbari M., Jabari A., Talebi A., Ashouri Movassagh S., Panahi Boroujeni A., Feizollahi N., Nikmahzar A., Pourahmadi M. (2020). Application of platelet-rich plasma (PRP) improves self-renewal of human spermatogonial stem cells in two-dimensional and three-dimensional culture systems. Acta Histochem..

[80] Moreno Aguilera P., Becerra Perdomo G.A., Martínez-Cano J.P., Figueroa Berríos M.L., Della Vedova F., Nakamura N., Sciarretta F.V., Lazzaretti Fernandes T., Marín Fermín T. (2025). Laws and regulations on platelet-rich plasma use for musculoskeletal pathologies in South America: A narrative review. Int. Orthop..

[81] Colomer-Selva R., Tvarijonavciute A., Franco-Martínez L., Hernández-Guerra Á.M., Carrillo J.M., Rubio M., Sopena J.J., Satué K. (2025). Physiological factors affecting platelet-rich plasma variability in human and veterinary medicine. Front. Vet. Sci..

[82] Mochizuki T., Ushiki T., Suzuki K., Sato M., Ishiguro H., Suwabe T., Edama M., Omori G., Yamamoto N., Kawase T. (2023). Characterization of leukocyte- and platelet-rich plasma derived from female college athletes: Growth factor, inflammatory cytokines, and anti-inflammatory cytokine levels. Int. J. Mol. Sci..

[83] Qin H., Yang H., Wang J., Jiang D., Chen Y., Wang W., Wang A. (2025). Progress in the clinical application of allogeneic platelet-rich plasma for diabetic foot treatment. Front. Endocrinol..

[84] Koller D., Beam A., Manrai A., Ashley E., Liu X., Gichoya J., Holmes C., Zou J., Dagan N., Wong T.Y. (2024). Why We Support and Encourage the Use of Large Language Models in NEJM AI Submissions. NEJM AI.

